# [^68^Ga]Ga-THP-tetrazine for bioorthogonal click radiolabelling: pretargeted PET imaging of liposomal nanomedicines†

**DOI:** 10.1039/d4cb00039k

**Published:** 2024-05-14

**Authors:** Aishwarya Mishra, Amaia Carrascal-Miniño, Jana Kim, Rafael T. M. de Rosales

**Affiliations:** a School of Biomedical Engineering & Imaging Sciences, King's College London St Thomas’ Hospital London SE1 7EH UK rafael.torres@kcl.ac.uk

## Abstract

Pretargeted PET imaging using bioorthogonal chemistry is a leading strategy for the tracking of long-circulating agents such as antibodies and nanoparticle-drug delivery systems with short-lived isotopes. Here, we report the synthesis, characterisation and *in vitro*/*vivo* evaluation of a new ^68^Ga-based radiotracer [^68^Ga]Ga-THP-Tetrazine ([^68^Ga]Ga-THP-Tz) for bioorthogonal click radiochemistry and *in vivo* labelling of agents with slow pharmacokinetics. THP-tetrazine (THP-Tz) can be radiolabelled to give [^68/67^Ga]Ga-THP-Tz at room temperature in less than 15 minutes with excellent radiochemical stability *in vitro* and *in vivo*. [^68^Ga]Ga-THP-Tz was tested *in vitro* and *in vivo* for pretargeted imaging of stealth PEGylated liposomes, chosen as a leading clinically-approved platform of nanoparticle-based drug delivery, and for their known long-circulating properties. To achieve this, PEGylated liposomes were functionalised with a synthesised transcyclooctene (TCO) modified phospholipid. Radiolabelling of TCO-PEG-liposomes with [^68/67^Ga]Ga-THP-Tz was demonstrated *in vitro* in human serum, and *in vivo* using both healthy mice and in a syngeneic cancer murine model (WEHI-164 fibrosarcoma). Interestingly *in vivo* data revealed that [^68^Ga]Ga-THP-Tz was able to *in vivo* radiolabel liposomes present in the liver and spleen, and not those in the blood pool or in the tumour. Overall, these results demonstrate the potential of [^68^Ga]Ga-THP-Tz for pretargeted imaging/therapy but also some unexpected limitations of this system.

## Abstract 2

A new radiotracer [^68^Ga]Ga-THP-tetrazine for bioorthogonal click radiochemistry is presented. Its ability for pretargeted imaging of long-circulating liposomal nanomedicines was tested *in vitro* and *in vivo* in both healthy and tumour murine models.

## Graphical abstract

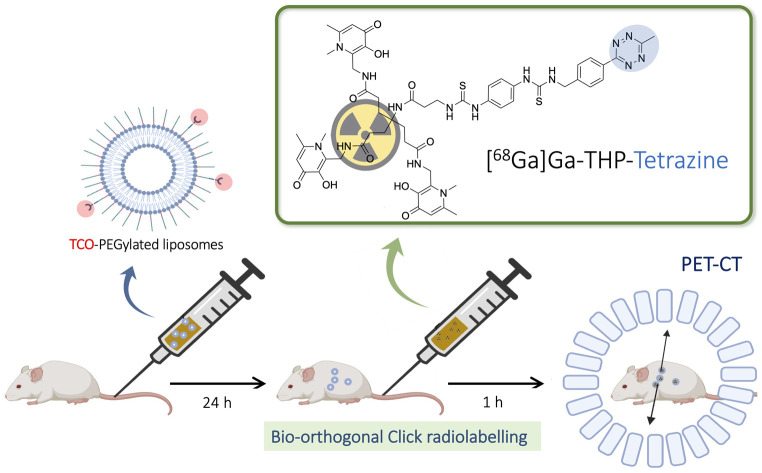

## Introduction

Bioorthogonal chemistry encompasses several chemical reactions that have minimal interaction and interference with a biological system, providing efficient chemical tools to create covalent bonds in intricate biological settings such as cells and living organisms.^1–3^ Recent advancements in bioorthogonal chemistry have allowed the development of new nuclear molecular imaging and therapy agents in the preclinical setting.^4^ These allow decoupled administration of a targeting vector followed by a separate administration of a radiolabelled tag with high affinity towards the former, and with fast pharmacokinetics and clearance. This approach has been named pretargeted imaging and differs substantially from the conventional nuclear imaging method of administering pre-radiolabelled vectors.^5^ One of its main advantages is the opportunity to visualise long-circulating and slow-clearing macromolecules (antibodies, nanomedicines, *etc.*) with short-lived PET radionuclides, which cannot be used with conventional imaging approaches.^2,6,7^ This helps in lowering radiation doses to the subject, which is a known issue for long-lived radiopharmaceuticals (*e.g.* radiohematotoxicities of radiolabelled mAb) and also provide high contrast, thanks to the fast clearance of the radiolabelled component. Pretargeting also minimises the structural modification required to radiolabel macromolecules, thereby minimising the effect of radiolabelling on their targeting properties. The pretargeting approach also has clear applications in radionuclide therapy, facilitating the delivery of therapeutic radionuclides to the target and minimising non-target radiation.

The most used bioorthogonal reactions for pretargeted imaging are strain-promoted azide–alkyne cycloaddition (SPAAC) and inverse electron demand Diels Alder reaction (IEDDA). The SPAAC reaction was first reported by Bertozzi *et al.* by introducing ring strain in the form of cyclooctyne, removing the need for Cu as a catalyst in previously reported Cu-catalysed azide–alkyne (3+2) cycloadditon.^3,8–10^ However, the SPAAC reaction has relatively slow reaction kinetics compared to the IEDDA approach reported by Blackman *et al.* in 2008.,^11^ making the latter the preferred option for pretargeted nuclear imaging.

**Fig. 1:**
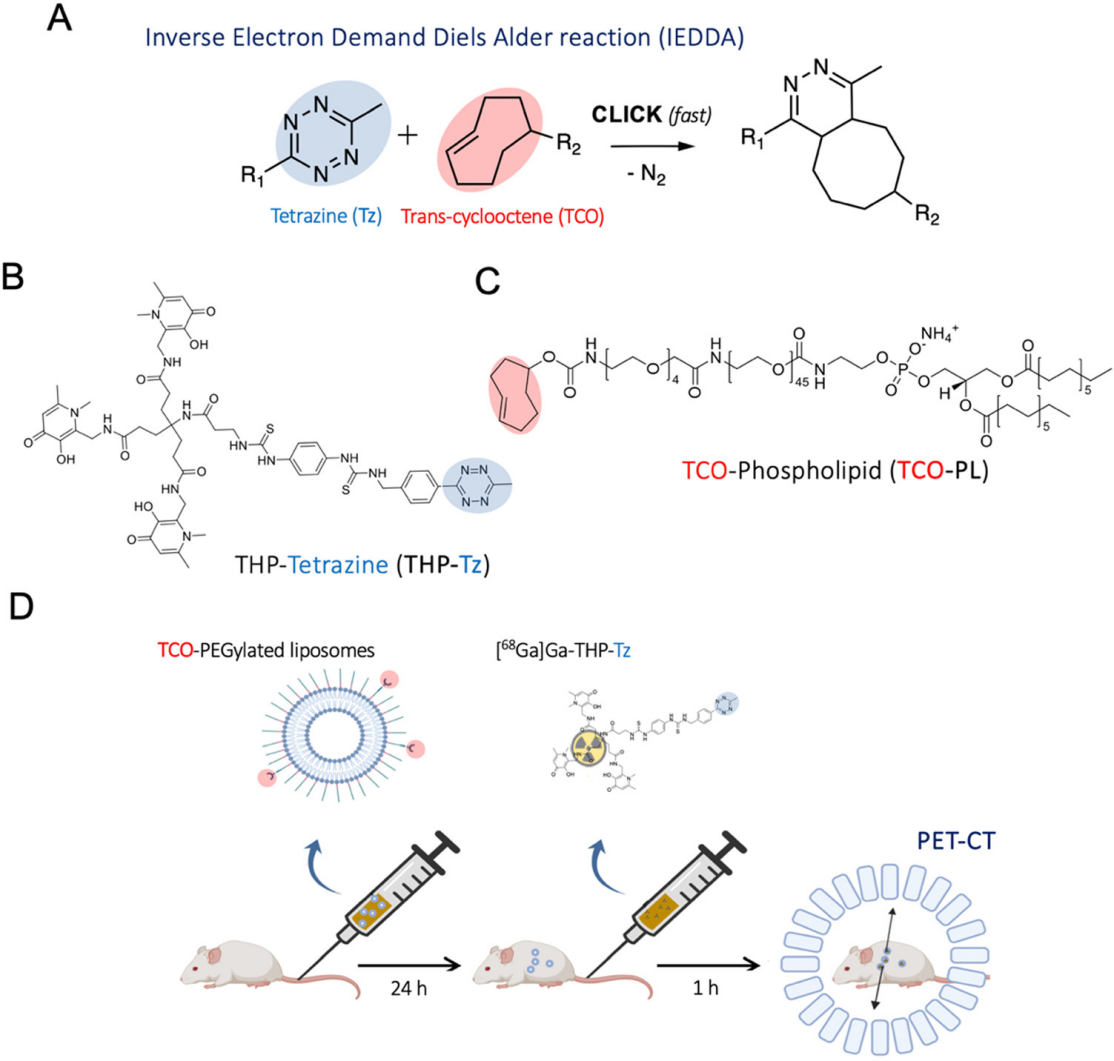
Components and schematics of the pretargeting study: (A) general scheme of inverse electron demand Diels Alder reaction (IEDDA) click reaction with fast reaction kinetics at physiological conditions where R_2_ is the biomolecule/antibody/nanomedicine being tracked and R_1_ is the nuclear/optical imaging agent; (B) and (C) chemical structure of the bioorthogonal pair used in this pretargeted imaging study: Ga-chelating tetrazine derivative THP-tetrazine (THP-Tz) (B) and bioorthogonal reactive phospholipid derivative TCO–PL (C); (D) schematic showing the reported procedure of *in vivo* pretargeted PET imaging of liposomal nanomedicines.

The IEDDA tetrazine ligation (Fig. 1(A)) shows superior reaction kinetics, with reported rate constants of up to 10^6–7^ M^−1^ s^−1^.^12^ In addition to this, its high specificity, the small molecule character, and the orthogonality towards biological moieties make this ligation highly attractive for pretargeting approaches *in vivo*. For nuclear imaging applications, the radiolabelled tag is created using the tetrazine component rather than the TCO since the latter has a susceptibility to undergo metabolic degradation when administered in trace amounts.^13^ IEDDA-based pretargeted nuclear imaging was first successfully validated *in vivo* in a preclinical setting with antibodies.^2^ Following this, several groups have performed various studies in mice for pretargeting of humanised antibodies such as A33, trastuzumab and cetuximab with PET radionuclides and, more recently, ^177^Lu for radiotherapy with excellent results showing high tumour uptake values comparable to conventional imaging methods with additional benefits such as low whole-body radiation dose.^14–21^

The PET radionuclide ^68^Ga (β^+^ 89%, *t*_1/2_ = 68 min, *E*_β+max_ = 1899 keV) has a shorter half-life than ^18^F and can be an excellent choice for pretargeted imaging due to its relatively low cost and convenient availability *via* benchtop ^68^Ge generators.^22^ However, limited studies have explored the potential of ^68^Ga labelled tetrazines for pretargeted imaging for antibodies or nanomedicines.^23^ We are interested in evaluating the use of ^68^Ga biorthogonal *in vivo* radiolabelling with clinically-relevant PEGylated liposomal nanomedicines. Our motivation is the need to better understand the *in vivo* behaviour of these nanosystems, to overcome issues such as EPR heterogeneity and potentially develop imaging-based patient stratification techniques to maximise their clinical efficacy.^24^ However, current *in vivo* PET imaging methods involve *ex vivo* radiolabelling and long-term tracking (due to the long biological half-life of PEGylated liposomes) which may result in significant radiation doses to patients.^25^ A pretargeted imaging approach may provide a solution to this, with potentially high sensitivity due to the large capacity of nanoparticles to carry reactive groups (*ca.* 10-fold higher reactive tags compared to antibodies).^26^ In addition, the same pretargeting imaging approach may support the development of theranostic pair nanomedicines by combined use of radiotherapy nuclides and PET diagnostic radionuclides, as already being explored with antibody-based targeting systems.

Here, we report a novel ^68^Ga-binding tetrazine (THP-tetrazine; Fig. 1) based on the highly efficient gallium chelator trishydroxypyridinone (THP) and its *in vitro*/*vivo* evaluation for pretargeted imaging of clinically relevant PEGylated liposomes. THP-tetrazine was synthesised, characterised, and radiolabelled with the gallium radionuclides ^68^Ga and ^67^Ga (*γ*, *t*_1/2_ = 78.3 h, 300 keV). The stability and biodistribution of the radiolabelled conjugate were determined *in vitro* and *in vivo*. To evaluate the potential of THP-tetrazine for both conventional radiolabelling and pretargeted imaging, a primary targeting vector – TCO-functionalised PEGylated liposomes (TCO-PEG-liposomes) – was also developed. The synthesised components of the pretargeting system were examined *in vitro* as well as *in vivo*. Initially, SPECT/CT images were obtained for directly radiolabelled ^67^Ga-THP-PEG-liposomes to provide the biodistribution of liposomes, prior to evaluation of [^68^Ga]Ga-THP-tetrazine for pretargeting of TCO-PEG-liposomes with PET/CT.

## Results and discussion

### Synthesis of 6-methyl tetrazine-THP (THP-Tz)

**Fig. 2:**
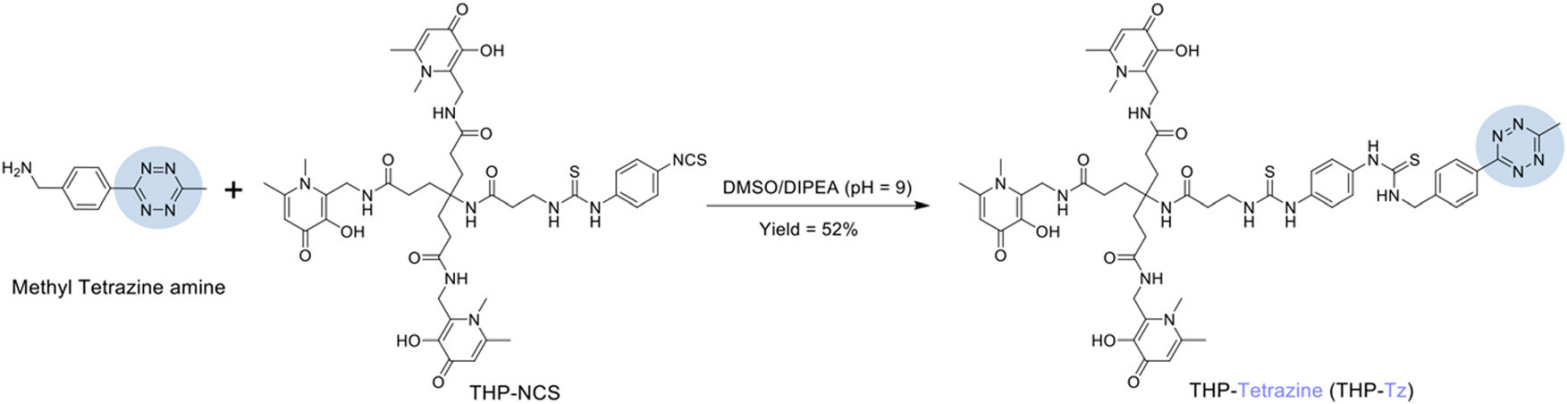
Reaction scheme for the synthesis of THP-Tz *via* reaction of methyl tetrazine amine and THP-isothiocyanate (THP-NCS).

THP-tetrazine was synthesised by a simple reaction of THP-Bz-SCN with 6-methyl tetrazine (Fig. 2). The chelator tris(hydroxypyridinone) (THP) was chosen due to its high affinity towards ^68^Ga^3+^ and fast complexation kinetics under mild conditions, compared to other gallium macrocyclic chelators such as DOTA.^27^ This allows single-step, purification-free radiolabelling.^28,29^ The amine derivative of methyl tetrazine has been widely used for conjugation with various imaging motifs and provides a straightforward method for conjugation with the amine-reactive THP-isothiocyanate. A 6-methyl tetrazine amine was chosen over the highly reactive hydrogen-substituted tetrazine due to its reported favourable stability and solubility in aqueous solution, which is a result of the stabilization effect of the electron-donating methyl group.^30^

The resulting thiourea linker has been successfully used in other THP-based radiotracers, and leads to stable compounds *in vivo*, based on several clinical and preclinical studies.^29,31–35^ The conjugation was performed at room temperature using DIPEA as a base to achieve a pH of 9–10 to keep the target amine on the 6-methyl tetrazine deprotonated. The mild conditions and aprotic solvent DMSO minimised the hydrolysis of THP-NCS to amine (*m*/*z* = 460) and degradation of tetrazine. The LCMS trace of the reaction mixture confirmed minimal side products as shown in Fig. S2 in the ESI.† These side products and unreacted starting material were removed using semi-preparative HPLC method 2. The characterisation data (MS and NMR spectroscopy) and purification methods of THP-Tz can also be found in the ESI.†

### Radiolabelling of THP-tetrazine

**Fig. 3:**
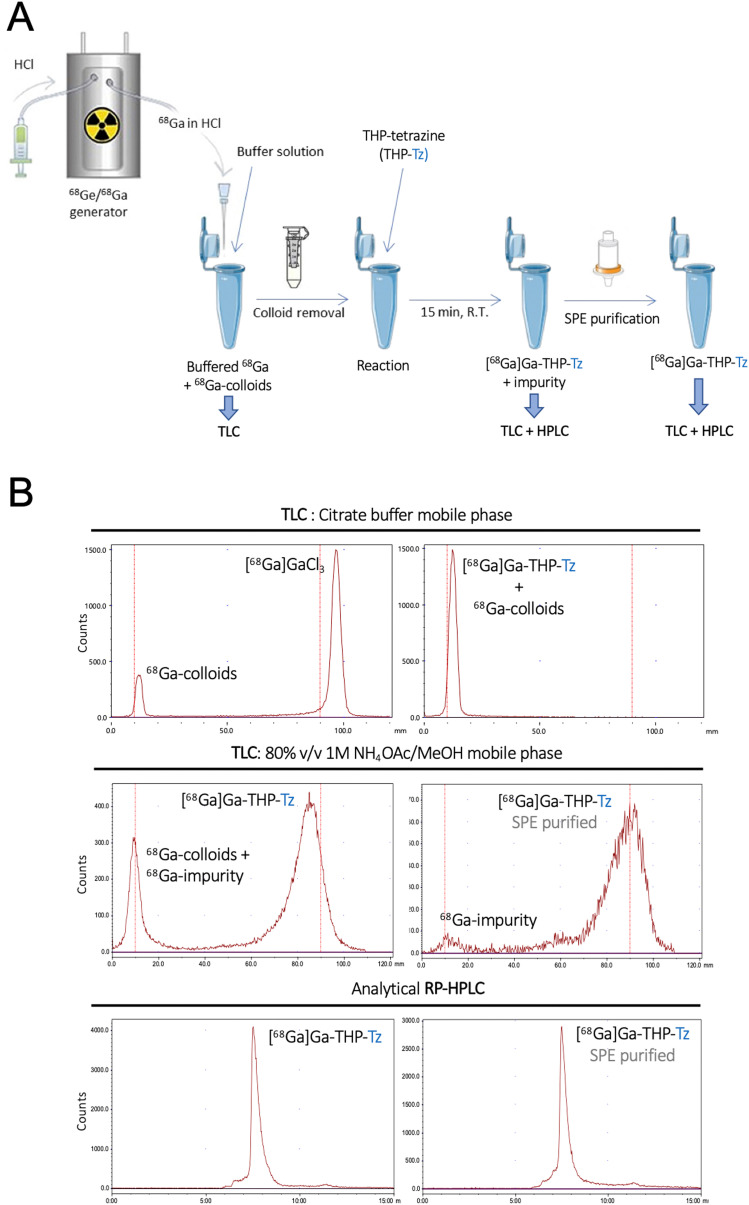
Synthesis, purification and characterisation of [^68^Ga]Ga-THP-Tz: (A) schematics of the radiolabelling of THP-tetrazine with generator produced ^68^Ga followed by purification and characterisation of the radiotracer; (B) radioTLC and radioHPLC chromatograms: top panel (left) neutralised ^68^Ga generator eluate on silica ITLC using citrate buffer; top panel (right) [^68^Ga]Ga-THP-Tz pre-purification on silica ITLC using citrate buffer; middle panel (left) [^68^Ga]Ga-THP-Tz pre-purification on silica ITLC using 80% v/v ammonium acetate/methanol; middle panel (right) SPE purified [^68^Ga]Ga-THP-Tz on silica ITLC using 80% v/v ammonium acetate/methanol (*R*_f_ = 0.8–1 for [^68^Ga]Ga-THP-Tz and *R*_f_ = 0 for lipophilic impurity); bottom panel (left) [^68^Ga]Ga-THP-Tz pre-purification characterised using analytical radioHPLC; bottom panel (right) SPE purified [^68^Ga]Ga-THP-Tz characterised by analytical radioHPLC.

The binding affinity of THP-Tz towards ^68^Ga was determined using radiolabelling experiments. THP-Tz was reacted with either ^68^Ga or ^67^Ga, and the radiolabelled products were characterised for purity and stability. The final optimised radiolabelling protocol is shown in Fig. 3(A). To achieve efficient radiolabelling it was considered important to ensure removal of colloidal ^68^Ga species that often form during the neutralisation of the ^68^Ga generator eluate (in 0.1 M HCl), as observed in the radioTLC chromatogram of neutralised ^68^GaCl_3_ using citrate buffer as mobile phase (Fig. 3(B) top left panel).^36^ Using this mobile phase, colloids are observed at *R*_f_ = 0 and, if observed, were efficiently removed during the pre-labelling purification using spin size-exclusion filtration. Successful removal of colloids from neutralised ^68^Ga was confirmed by radio-TLC (Fig. S6, ESI†). Removal of colloidal species is particularly relevant when evaluating nanoparticulates *in vivo*, as they share similar accumulation/excretion organs (*e.g.* liver, spleen). The radiolabelling reaction was found to be fast at mild conditions, in agreement with other THP compounds.^28,29,37^ In a typical reaction, THP-Tz (5 μg, 1 mg mL^−1^) was incubated with sodium carbonate (1 M, pH 6)-neutralised purified ^68^Ga for 15 minutes followed by analysis by radio-TLC and radio-HPLC (Fig. 3(B)), indicating the formation of a single radiolabelled species. The radio-HPLC and radioTLC (citrate buffer) of the reaction showed a single radioactive peak corresponding to [^68^Ga]Ga-THP-Tz (radio-TLC in citrate buffer: *R*_f_ = 0, radio-HPLC *t*_r_ = 8 min) and the absence of free ^68^Ga (*R*_f_ = 1).

However, we found that a large fraction of the radioactivity after [^68^Ga]Ga-THP-Tz synthesis was often found irreversibly bound to glass vials and formed an impurity which could not be detected by previous characterisation methods (radioHPLC and radioTLC in citrate buffer). A different method of radio-TLC in ammonium acetate : methanol (1 M, 80% v/v) showed the presence of the impurity at *R*_f_ = 0 and [^68^Ga]Ga-THP-Tz at *R*_f_ = 1, which was successfully removed using SEP-PAK C18 purification followed by characterisation using radioTLC and radioHPLC (Fig. 3(B) middle panel: radio-TLC and bottom panel: radio-HPLC). The nature of this insoluble impurity that binds irreversibly to glass surfaces remains unknown despite our efforts to characterise it. It is worth noting that it has not been observed with other THP-based small molecule radiotracers with thiourea linkers,^29,31,32,34,35^ strongly suggesting it is specific to this tetrazine conjugate. The purified [^68^Ga]Ga-THP-Tz (RCY = 45 ± 5%, Apparent *A*_m_ ≈ 2.2 GBq μmol^−1^) eluted in pure methanol was subsequently dried under nitrogen and resuspended in 5% DMSO/water used for further *in vitro* and *in vivo* validation. [^67^Ga]Ga-THP-Tz was prepared using the same procedure. In contrast to other THP-based radiotracers, the requirement of the extra SPE purification step in the radiolabelling of THP-tetrazine decreases the ease of the well-known THP radiochemistry. However, the exceptionally high affinity of THP towards Ga at room temperature allows the reaction to take place at nanomole concentrations of the THP-tetrazine which is lower or comparable to the concentration required for other Ga chelators.^27^

The partition coefficient in water (log *P*) and pH 7.4 buffer (log *D*_7.4_) of [^68^Ga]Ga-THP-Tz was −0.47 ± 0.01 and −0.44 ± 0.03, respectively. These values are consistent with a significantly higher lipophilic character compared to existing ^68^Ga labelled conjugates of THP (log *D*_7.4_ [^68^Ga]Ga-THP-Pam = −2.72 ± 0.07; log *D*_7.4_ [^68^Ga]Ga-THP-PSMA = −5.4 ± 0.1).^28^ and could be explained by the lack of low p*K*_a_ groups in [^68^Ga]Ga-THP-Tz. The log *D*_7.4_ of [^68^Ga]Ga-THP-Tz is also higher than other tetrazine-based ^68^Ga imaging tracers such as [^68^Ga]Ga-HBED-CC-PEG_4_-Tz = −1.28 ± 0.19 and [^68^Ga]Ga-DOTA-PEG_4_-Tz = −1.45 (both H-tetrazines), [^68^Ga]Ga-DOTA-PEG_11_-Tz = −2.19 ± 0.03^23,38^ and [^68^Ga]Ga-DOTA-GA-tetrazine = −2.3 (methyl-tetrazine).^39^ The relative high log *D*_7.4_ of [^68^Ga]Ga-THP-Tz compared with these Tz conjugates could be explained by the lack of PEG units and ionisable carboxylic acid groups, and is consistent with a high lipophilic character which is known to result in slow pharmacokinetic clearance and a preference for hepatobiliary excretion and uptake in the liver, gall bladder and intestines.^40–42^

The radiochemical stability of [^68^Ga]Ga-THP-Tz was evaluated by incubation in human serum at 37 °C for 3 hours and size-exclusion (SE) chromatography (HPLC). SE-HPLC efficiently separated [^68^Ga]Ga-THP-Tz and serum components, providing a useful method to assess demetallation *ex vivo*. This was considered an important test as blood serum proteins such as transferrin and lactoferrin are known to transchelate gallium from other bioconjugates *in vivo*. The results showed minimal transchelation of ^68^Ga to blood serum proteins and a high stability of >95% after 3 hours of incubation (Fig. S7 in ESI†) and supporting the transition to evaluate [^68^Ga]Ga-THP-Tz *in vivo.*

### *In vivo* imaging and biodistribution of [^68^Ga]Ga-THP-Tz in healthy and tumour-bearing mice

**Fig. 4:**
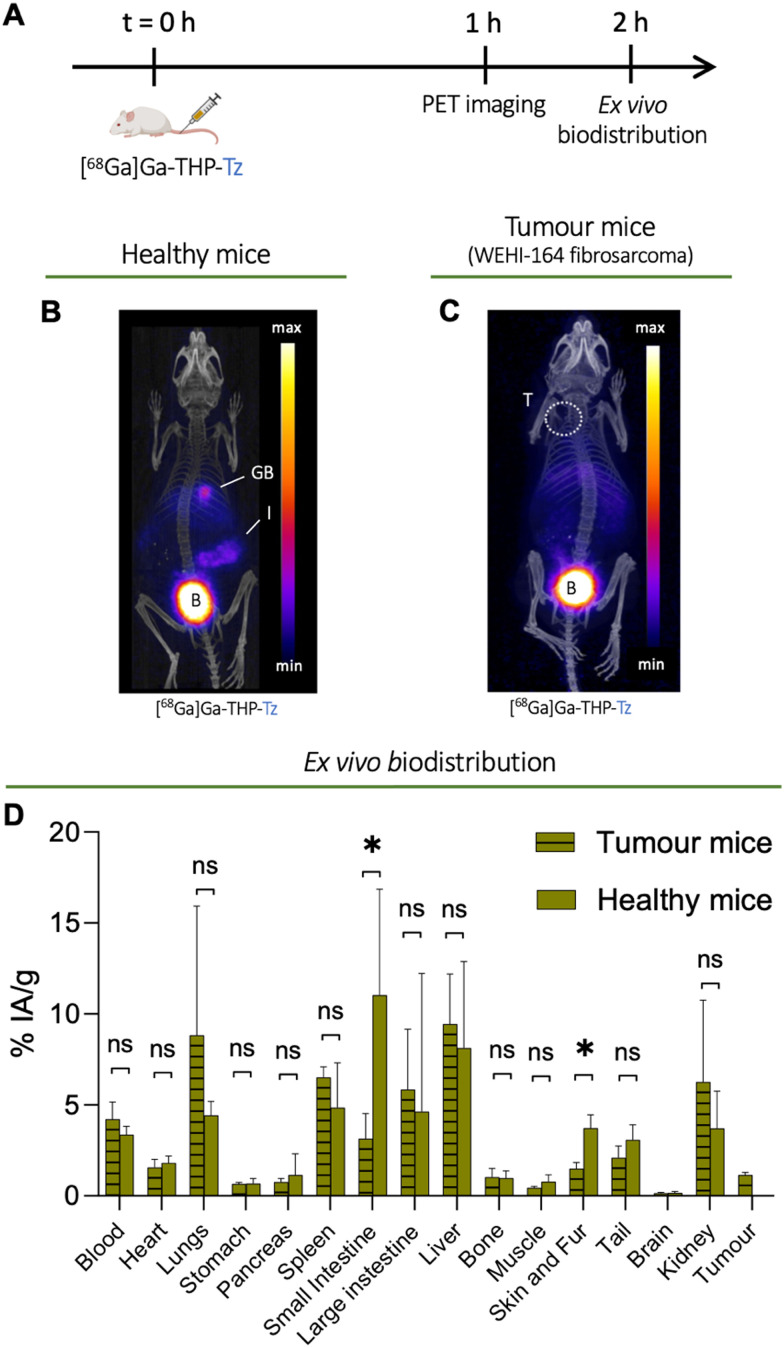
PET imaging and biodistribution of [^68^Ga]Ga-THP-tetrazine ([^68^Ga]Ga-THP-Tz, 2–5 MBq per mouse) in healthy mice and fibrosarcoma WEHI-164 tumour-bearing mice. (A) *In vivo* imaging protocol for the PET and *ex vivo* biodistribution study of the small molecule tracer [^68^Ga]Ga-THP-Tz; (B) PET-CT image of representative healthy mouse administered with [^68^Ga]Ga-THP-Tz at 1 h p.i.; GB = gall bladder; I = intestines; B = urinary bladder; (C) PET-CT image of fibrosarcoma WEHI-164 tumour (T) bearing mice administered with [^68^Ga]Ga-THP-Tz at 1 h p.i. (dotted circle shows the tumour region); (D) *ex vivo* biodistribution at 2 h p.i. in healthy and tumour-bearing mice reported in %IA g^−1^ units (*n* = 4 and 3, respectively).

To study the biodistribution of [^68^Ga]Ga-THP-Tz, we designed an *in vivo* experiment using preclinical PET imaging (Fig. 4(A)). Both healthy and tumour-bearing mice (mouse fibrosarcoma cell line WEHI-164) were imaged 1 h after intravenous injection of [^68^Ga]Ga-THP-Tz (Fig. 4(B) and (C)). This was followed by *ex vivo* biodistribution quantification of organ uptake performed post-imaging (Fig. 4(D)). These two groups served as negative controls to the pretargeting imaging studies (*vide infra*). Due to its small size and relatively lipophilic nature (*vide supra*), [^68^Ga]Ga-THP-Tz was expected to clear through both hepatobiliary and renal excretion pathways. The PET images (Fig. 4(B) and (C)) show high uptake in the urinary bladder, gall bladder, and intestines which is consistent with mixed hepatobiliary/renal clearance. Relatively low uptake was observed in the blood (<5% IA g^−1^; IA = injected activity) due to fast clearance. The high uptake in the urinary bladder and gall bladder was expected due to the clearance of the imaging agent *via* renal and hepatobiliary excretion pathways, respectively. In the fibrosarcoma model, there was very low tumour uptake (1.1 ± 0.1% IA g^−1^; IA = injected activity) most likely due to remaining activity in the blood pool (4.2 ± 0.9% IA g^−1^). From the biodistribution and image quantification data (Fig. 4(D)), high uptake was measured in the lungs (8.8 ± 7.1% IA g^−1^), liver (9.4 ± 2.7% IA g^−1^), and spleen (6.5 ± 0.6% IA g^−1^). The high lung uptake values observed could be explained by its lipophilic nature potentially leading to a certain level of aggregation of THP-Tz due to hydrophobic interaction with plasma components. It should be noted, however, that no aggregation was observed during our *in vitro* stability studies in human serum (*vide supra*).

### Synthesis of TCO-PEG-liposomes

**Fig. 5:**
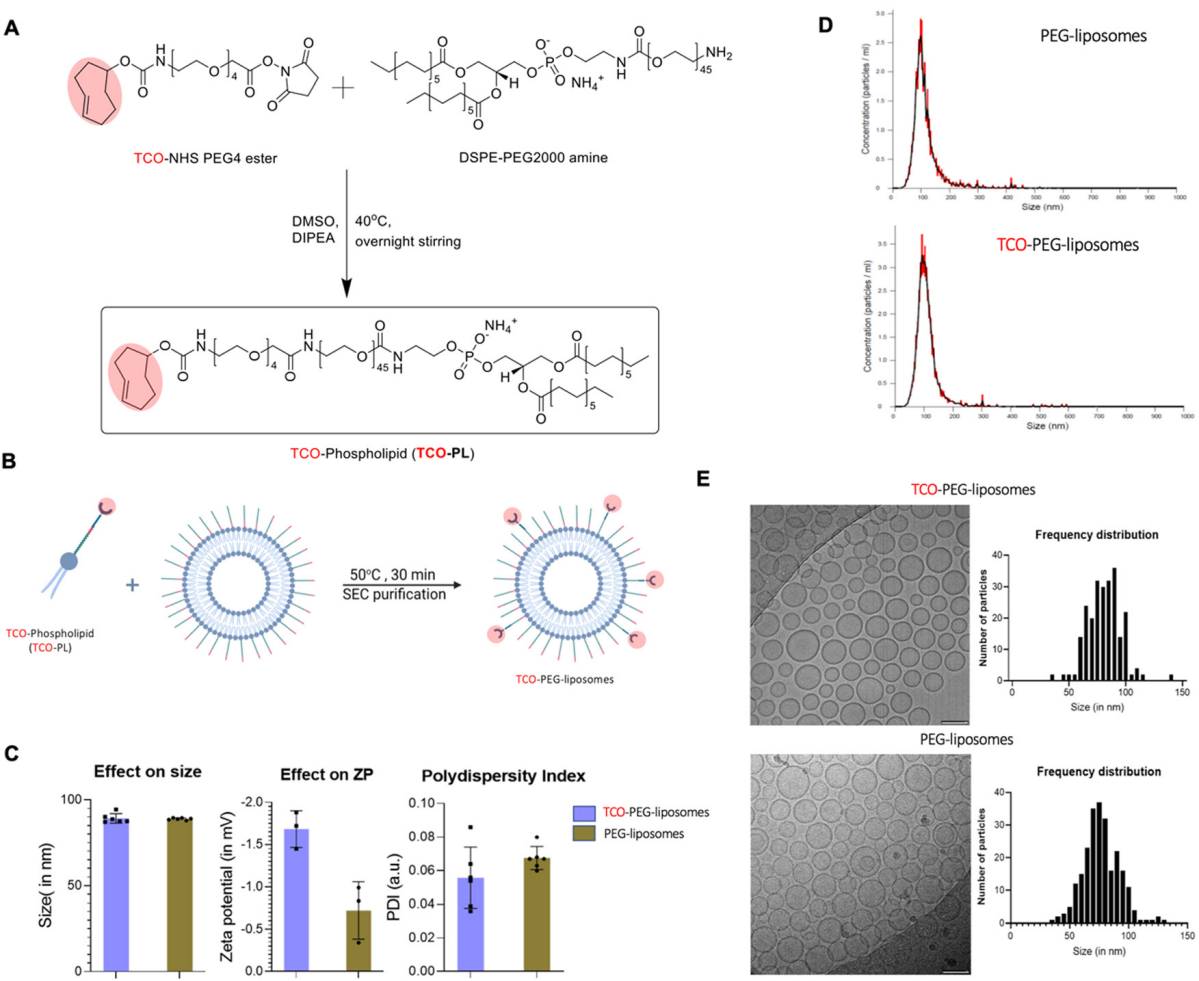
Synthesis and characterisation of modified TCO-PEG-liposomes. (A) Synthesis of TCO–phospholipid (TCO–PL): DSPE-PEG2000 amine was reacted with NHS ester derivative of TCO to give the phospholipid–TCO conjugate (TCO–PL); (B) synthesis of TCO-PEG-liposomes: insertion of TCO–PL conjugate into the lipid bilayer of preformed PEGylated liposomes at mild heating below phase transition temperature; (C) characterisation of TCO-PEG-liposomes using dynamic light scattering (DLS): no major impact was observed on hydrodynamic size, zeta potential and polydispersity index post modification of PEGylated liposomes; (D) characterisation of TCO-PEG-liposomes using nanoparticle tracking analysis (NTA): (left) PEG(2k)-liposomes (concentration: 2.88 × 10^16^ ± 2.17 × 10^15^ particles per mL, mode: 97.8 nm, mean: 116.2 nm), (right) TCO-PEG-liposomes (concentration: 3.74 × 10^15^ ± 2.36 × 10^14^ particles per mL, mode: 93.1 nm, mean: 110.6 nm); (E) characterisation of TCO-PEG-liposomes using Cryo-electron microscopy: (top) electron micrograph showing TCO-PEG-liposomes have retained their spherical nature and the size distribution is not altered by insertion of TCO–PL into the bilayer of liposomes and bilayer nature is also retained, (bottom) electron micrograph showing PEG(2k)-liposomes spherical morphology and bilayer structure.

PEGylated liposomes were chosen as the targeting vector as a leading platform of drug delivery in a clinical setting (*e.g.* Doxil/Caelyx®). Furthermore, being able to image them in a pretargeted manner could help us unlock their full theranostic potential. To create TCO containing liposomes, we firstly synthesised TCO-DSPE-PEG(2000) (TCO–PL), a phospholipid required to functionalise the surface of PEGylated liposomes and provide them with bioorthogonal reactivity. TCO–PL was synthesised by the reaction between the NHS ester derivative of TCO (*trans*-cyclooctene-PEG_4_-NHS) and the amine derivative of the phospholipid 1,2-stearoyl-*sn-glycerol*-3-phosphoethanolamine-*N*-amino (polyethylene glycol)-2000 as the ammonium salt (DSPE-PEG(2000)-amine) (Fig. 5(A)). The PEG spacer in TCO-NHS allows for increased ease of handling by providing water solubility, and an increased distance between the amine-containing compound (here, phospholipid) to be modified, and the reactive alkene. The 2000 Da length PEG linker in the DSPE-PEG2000 amine also plays an important role by allowing accessibility of TCO molecules to the surface of long-circulating PEGylated liposomes. The presence of the TCO–PL in the product was confirmed by comparison of the mass spectra of the TCO–PL and DSPE-PEG2000 amine. A clear shift was observed for the *z* = 1 species towards the higher molecular weight from DSPE-PEG-amine ([M + 2H]^+^ observed *M*_W_ = 2774, calculated *M*_W_ = 2774) to TCO–PL ([M + H]^+^ observed *M*_W_ = 3305, calculated *M*_W_ = 3305). The characterization of the synthesised TCO–PL using NMR and mass spectrometry confirmed the purity and expected structure, confirming the synthesis of TCO–PL in high yield and purity (see ESI† for MS and NMR characterisation).

To obtain the targeting vector TCO-PEGylated liposomes (Fig. 1(D)), we performed insertion of the synthesised TCO–PL in the bilayer of the PEGylated liposomes. Insertion was achieved using a protocol previously shown to allow insertion of folate targeting phospholipids without significant modification of the original properties of PEGylated liposomes (Fig. 5(B)).^43^ The insertion process occurs at an elevated temperature which is just below the phase transition temperature of HSPC (58 °C) to increase the fluidity of the membrane for the incorporation of the external phospholipids. The final insertion conditions chosen were incubation temperature: 50 °C, incubation time: 30 min, and TCO–PL relative concentration: 5 mol%. The PEGylated liposomes used were chosen for having the same physicochemical properties as stealth liposomes extensively used in the clinic such as Doxil/Caelyx® (size: 89.4 ± 0.6 nm, concentration: 60.0 ± 0.9 mM, PDI: 0.03 ± 0.01 measured by DLS). The product (TCO-PEGylated liposomes) was purified by size exclusion (PD Minitrap™ G-25 size exclusion column) and analysed *via* dynamic light scattering (DLS; Fig. 5(C)), nanoparticle tracking analysis (NTA; Fig. 5(D)), and cryo-transmission electron microscopy (Cryo-TEM; Fig. 5(E)). The insertion was considered successful as the physicochemical properties of TCO-PEG-liposomes were the same as PEG(2k)-liposomes, and the TCO-PEG-liposomes had gained bioorthogonal reactivity towards tetrazine to undergo cycloaddition at high reaction rates (*vide infra*). The DLS studies (Fig. 5(C)) confirmed that surface modification had no impact on the hydrodynamic size of the PEGylated liposomes. The *z*-potential of the TCO-PEG-liposomes decreased by *ca.* 1 mV compared to PEGylated liposomes and remained within the range of other PEGylated stealth liposomes (Fig. 5(C)). The NTA performed on the TCO-PEG-liposomes and unmodified liposomes confirmed modification had no impact on the size distribution, and polydispersity (Fig. 5(D)). Similarly, cryo-electron microscopy of TCO-PEG-liposomes reaffirmed the previous observations about the impact of the insertion reaction on the size and size distribution (Fig. 5(E)). Moreover, the cryo-electron micrographs confirmed no modification to the bilayer nature of the liposomes post-incorporation of TCO–PL on the surface.

### ^67/68^Ga Radiolabelling of TCO-PEG-liposomes

The directly labelled liposomes ^67/68^Ga-THP-PEG-liposomes were synthesised by incubation of radiolabelled [^67/68^Ga]Ga-THP-Tz with TCO-PEG-liposomes for 15 minutes providing ^67^Ga-THP-PEG-liposomes and ^68^Ga-THP-PEG-liposomes with a radiochemical yield of 65 ± 9% and 74 ± 6%, respectively (*A*_m_ = 1 MBq μmol^−1^; *A*_s_ = 1 MBq/10^13^ particles); after size exclusion chromatography purification. The highly reproducible labelling was achieved in as low as 15 minutes and is desirable for labelling of liposomes with short lived isotopes. The incubation was performed under continuous shaking at 37 °C providing ideal conditions for testing the bioorthogonal reaction. Any unreacted [^67/68^Ga]Ga-THP-Tz was retained in the size exclusion column allowing for quantitative measurement of liposome radiolabelling. When ^67^Ga-THP-PEG-liposomes were incubated in human serum, high radiochemical stability was observed (91 ± 4%) after 48 h of incubation at 37 °C.

### *In vivo* imaging and biodistribution of [^67^Ga]Ga-THP-PEG-liposomes

**Fig. 6:**
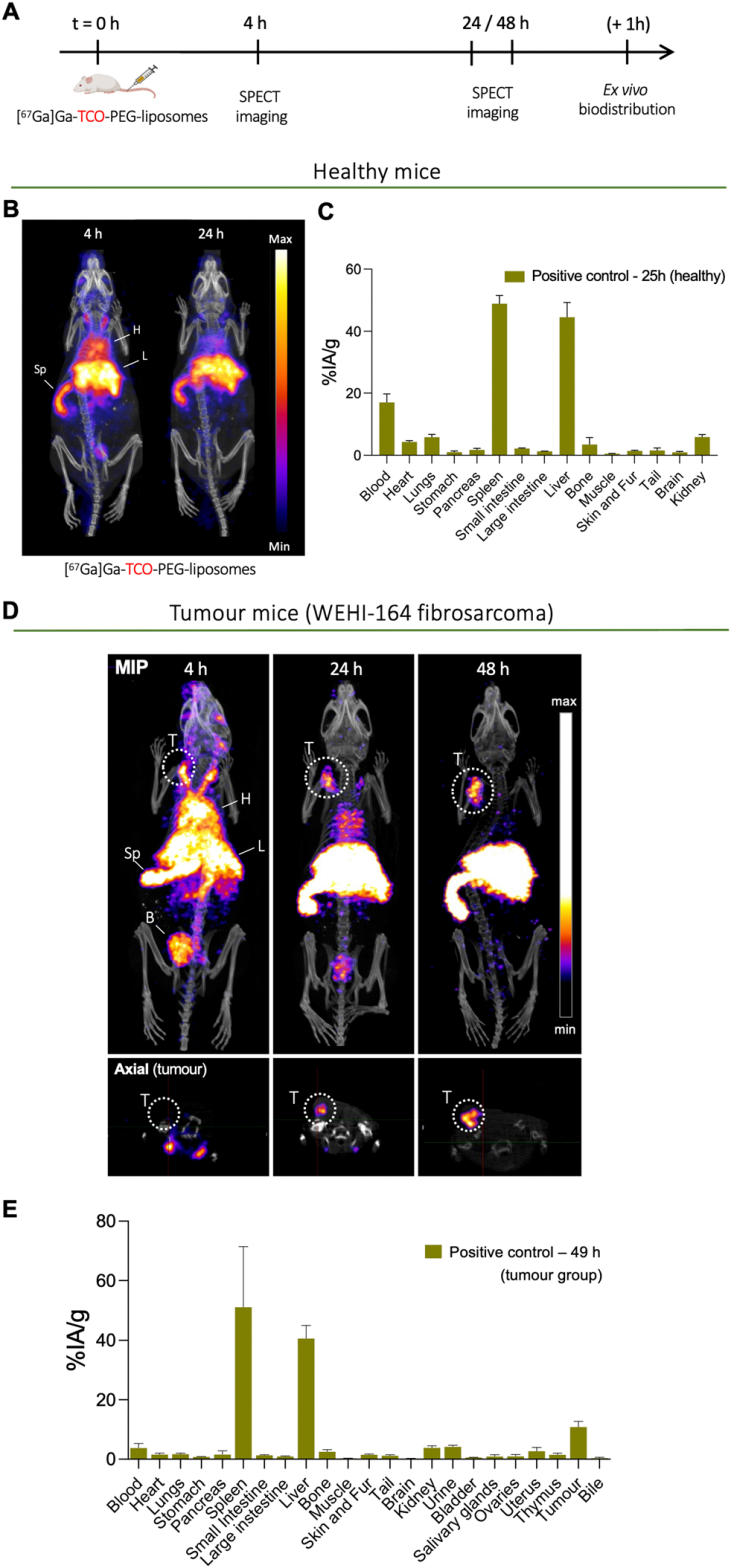
SPECT imaging and biodistribution of directly radiolabelled PEGylated liposomes using bioorthogonal chemistry ([^67^Ga]Ga-THP-PEG-liposomes, 3 MBq per mouse) in healthy and fibrosarcoma WEHI-164 tumour mice (positive control): (A) *in vivo* imaging protocol for SPECT and biodistribution studies with [^67^Ga]Ga-THP-PEG-liposomes; (B) SPECT-CT image of a representative healthy mouse administered with [^67^Ga]Ga-THP-PEG-liposomes at *t* = 4 h and *t* = 24 h p.i.; (C) *ex vivo* biodistribution at *t* = 25 h (*n* = 4); (D) top: maximum intensity projections of SPECT-CT image of a representative WEHI-164 tumour-bearing mouse administered with [^67^Ga]Ga-THP-PEG-liposomes at 4, 24, 48 h p.i. (H = heart; Sp = spleen; L = liver; T = tumour); bottom: axial section of SPECT images showing tumour cross-section at different time points, tumour highlight within dotted circular lines; (E) *ex vivo* biodistribution in the WEHI-164 tumour bearing mice at *t* = 49 h reported in %IA g^−1^ (*n* = 4).

To determine the biodistribution and pharmacokinetics of long-circulating PEGylated liposomes, TCO-PEG-liposomes were radiolabelled with long-lived radionuclide ^67^Ga (*t*_1/2_ = 78.3 h) and administered intravenously into both healthy and tumoured mice (mouse fibrosarcoma cell line WEHI-164). Mice were then imaged by SPECT imaging, followed by *ex vivo* organ biodistribution studies after the imaging studies (Fig. 6(A)). The *in vivo* imaging of directly labelled [^67^Ga]Ga-THP-PEG-liposomes also represents the positive control group for the pretargeting studies (*vide infra*). Henceforth the direct imaging of [^67^Ga]Ga-THP-PEG-liposomes will be addressed as the positive control group in this work.

The positive control group in healthy mice involved the intravenous administration of [^67^Ga]Ga-THP-PEG-liposomes, followed by SPECT-CT imaging at two-time points (*t* = 4, 24 h) and *ex vivo* biodistribution at *t* = 25 h. The SPECT-CT images (Fig. 6(B)) show the expected biodistribution of stealth PEGylated liposomes with a high amount of liposomes circulating in the blood pool at the early timepoint and increasing uptake in spleen and liver over time. This *in vivo* behaviour is common for PEGylated liposomal nanomedicines.^44^

The positive control group in tumoured mice involved intravenous administration of [^67^Ga]Ga-THP-PEG-liposomes followed by SPECT-CT imaging at *t* = 4, 24, 48 h (*n* = 4) and *ex vivo* biodistribution post imaging at *t* = 48 h (*n* = 3). The multiple imaging time points were chosen to determine the pharmacokinetics of [^67^Ga]Ga-THP-PEG-liposomes and accumulation at the tumour site over time, and importantly, inform the pretargeting experiments. Similar to the healthy control group (*vide supra*), the SPECT-CT images (Fig. 6(D)) also show the expected *in vivo* behaviour of PEGylated liposomes, with high levels of blood-circulating [^67^Ga]Ga-THP-PEG-liposomes in the first 24 h, and a gradual increase in uptake up to 48 h in the liver (40.7 ± 4.4% IA g^−1^), spleen (51.0 ± 20.4% IA g^−1^), and tumour (10.8 ± 1.9% IA g^−1^) as a result of the EPR effect. This biodistribution at later timepoint was also confirmed by *ex vivo* gamma-counting (Fig. 6(E)). Radioactivity was also observed in the urinary bladder at early timepoints, presumably due to renal clearance of a minor proportion of small liposomes/micelles, as observed with other liposomal systems.^45^

**Fig. 7:**
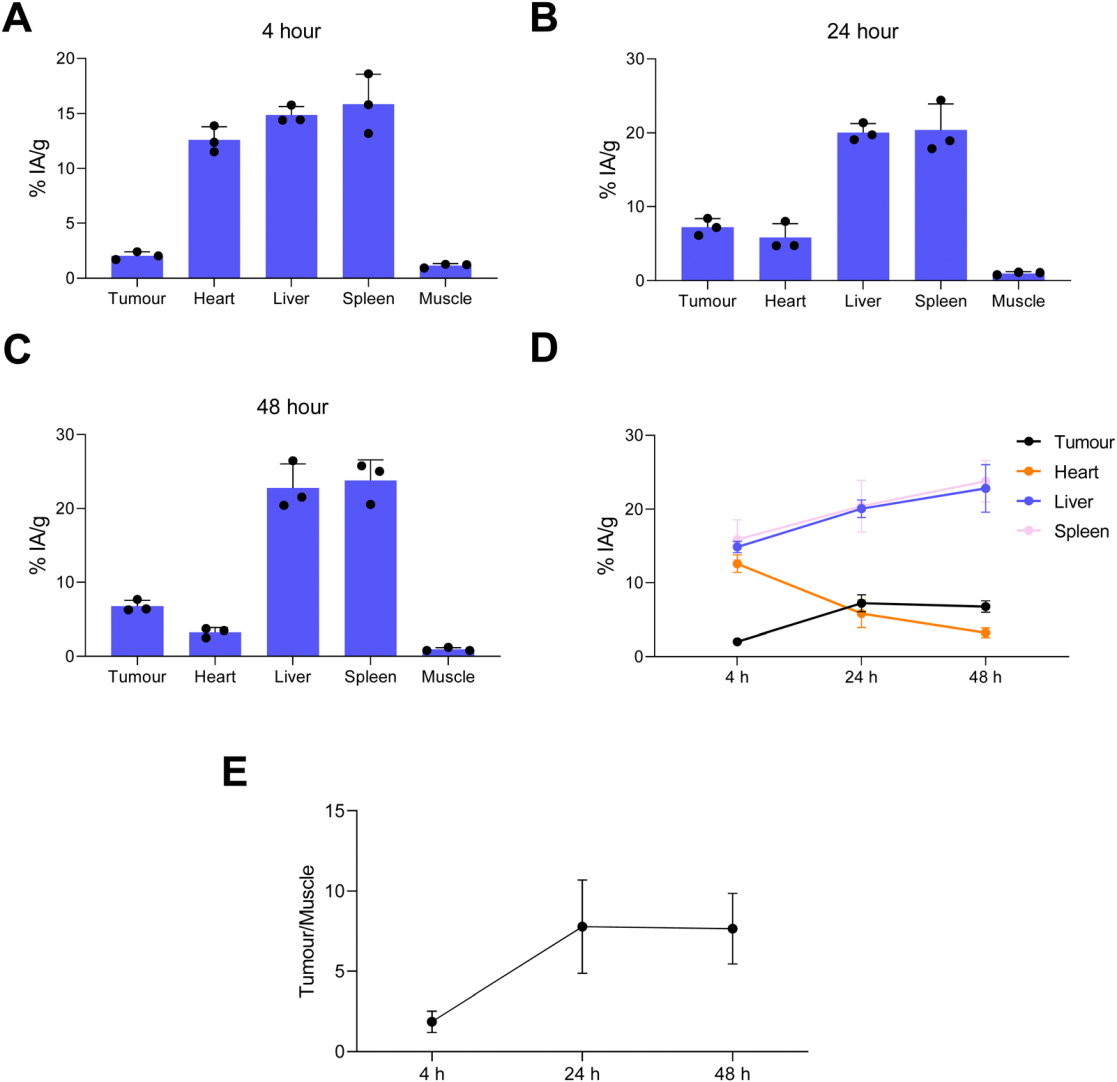
Image-based analysis of the positive control group SPECT images ([^67^Ga]Ga-THP-PEG-liposomes) in the fibrosarcoma (WEHI-164) tumour model: tissue accumulation of radiolabelled liposomes [^67^Ga]Ga-THP-PEG-liposomes in tumour, heart, liver, spleen and muscle at different time points (*n* = 4); (A) 4 h, (B) 24 h, (C) 48 h; (D) data from A–C plotted as a time-activity graph; (E) variation of tumour to muscle ratio (T/M) contrast over different time points of scanning. (% IA g^−1^ values).

Image quantification of the SPECT images was performed (Fig. 7). This was in agreement with the *ex vivo* biodistribution data showing liposomes in the blood pool, tumour, liver and spleen with decreasing levels in blood with increasing time (Fig. 7(E)). The maximum tumour/muscle contrast was reached between 24–48 h (Fig. 7(F)). Overall this SPECT imaging study of directly labelled liposomes ([^67^Ga]Ga-THP-PEG-liposomes) showed the expected pharmacokinetics and biodistribution PEGylated liposomes, reaching maximum tumour uptake and tumour/muscle ratios at 24 h. This data strongly suggested *t* = 24 h as the optimal timepoint to target tumour-accumulated liposomes *in vivo* using the pretargeted approach (*vide infra*)

### Pretargeting experiments

#### *In vitro* experiments

**Fig. 8:**
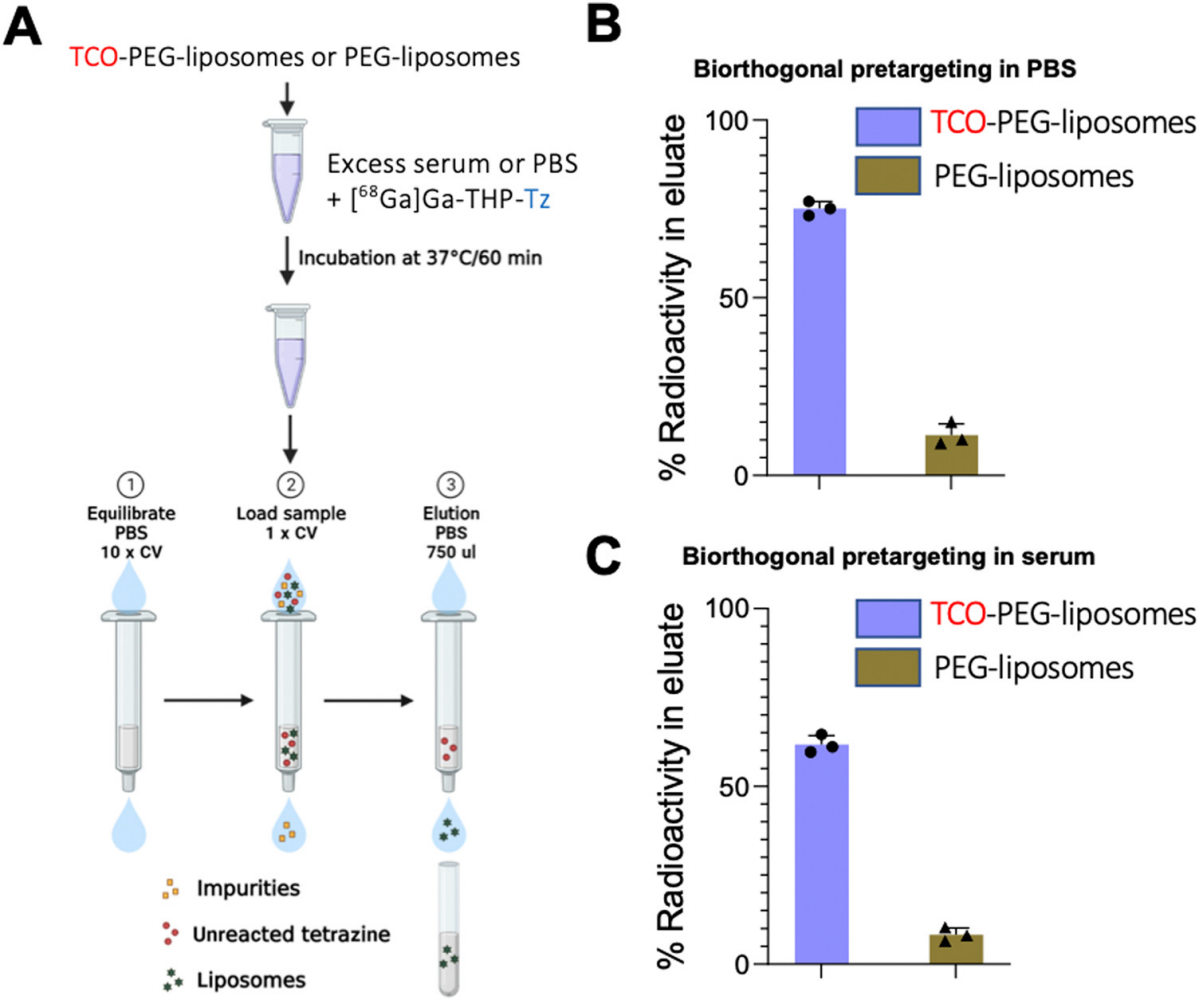
*In vitro* bioorthogonal pretargeting of TCO-PEG-liposomes: (A) schematic of the *in vitro* bioorthogonal pretargeting performed to validate the TCO/tetrazine pretargeting system. Two different *in vitro* bioorthogonal pretargeting experiments were performed where TCO-PEG-liposomes were incubated in either PBS or serum followed by the addition of [^68^Ga]Ga-THP-Tz and incubation at 37 °C for 30 minutes. The incubated samples were then purified *via* size exclusion chromatography and radioactivity attached to the TCO-PEG-liposomes was determined. Unmodified PEG-liposomes were tested as controls; (B) bioorthogonal pretargeting in PBS showing 75 ± 2% (*n* = 3) labelling of TCO-PEG-liposomes (*n* = 3); (C) bioorthogonal pretargeting in serum showing 62 ± 3% (*n* = 3) labelling of TCO-PEG-liposomes.

Following the synthesis of all the components of the pretargeting system and successful direct radiolabelling of liposomes *via* bioorthogonal chemistry, we tested the bioorthogonal pretargeting *in vitro.* This was done both under high dilution conditions (in PBS) and in the presence of blood serum proteins (human serum) to simulate the *in vivo* conditions. This involved pre-incubation of TCO-PEG-liposomes (200 μL, 4 mM) with human serum (500 μL) or PBS (500 μL) for 1 h at 37 °C, followed by the addition of [^68^Ga]Ga-THP-Tz (100 μL, 1–4 MBq) for 30 min at 37 °C under continuous agitation. Size exclusion chromatography was used in both cases to isolate and quantify liposome-bound ^68^Ga from that bound to serum components (Fig. 8(A)). These experiments closely resemble our proposed *in vivo* bioorthogonal pretargeting approach and the results showed successful radiolabelling with a high efficiency of 75 ± 2% (*n* = 3) in PBS dilution conditions (Fig. 8(B)). In human serum, TCO-PEG-liposomes were radiolabelled with an efficiency of 62 ± 3% (*n* = 3) (Fig. 8(C)). These results further confirmed the high reactivity of the liposome-embedded TCO towards tetrazine within a biologically relevant environment and supported the *in vivo* evaluation of this approach. Furthermore, the high radiolabelling of TCO-PEG-liposomes with [^68^Ga]Ga-THP-Tz in the presence of serum proteins strongly suggests that the TCO group has not isomerised into its unreactive CCO isomer, a possibility that has been observed with antibody pretargeting systems.^46^

### *In vivo* pretargeting of TCO-PEG-liposomes in healthy and tumoured mice

**Fig. 9:**
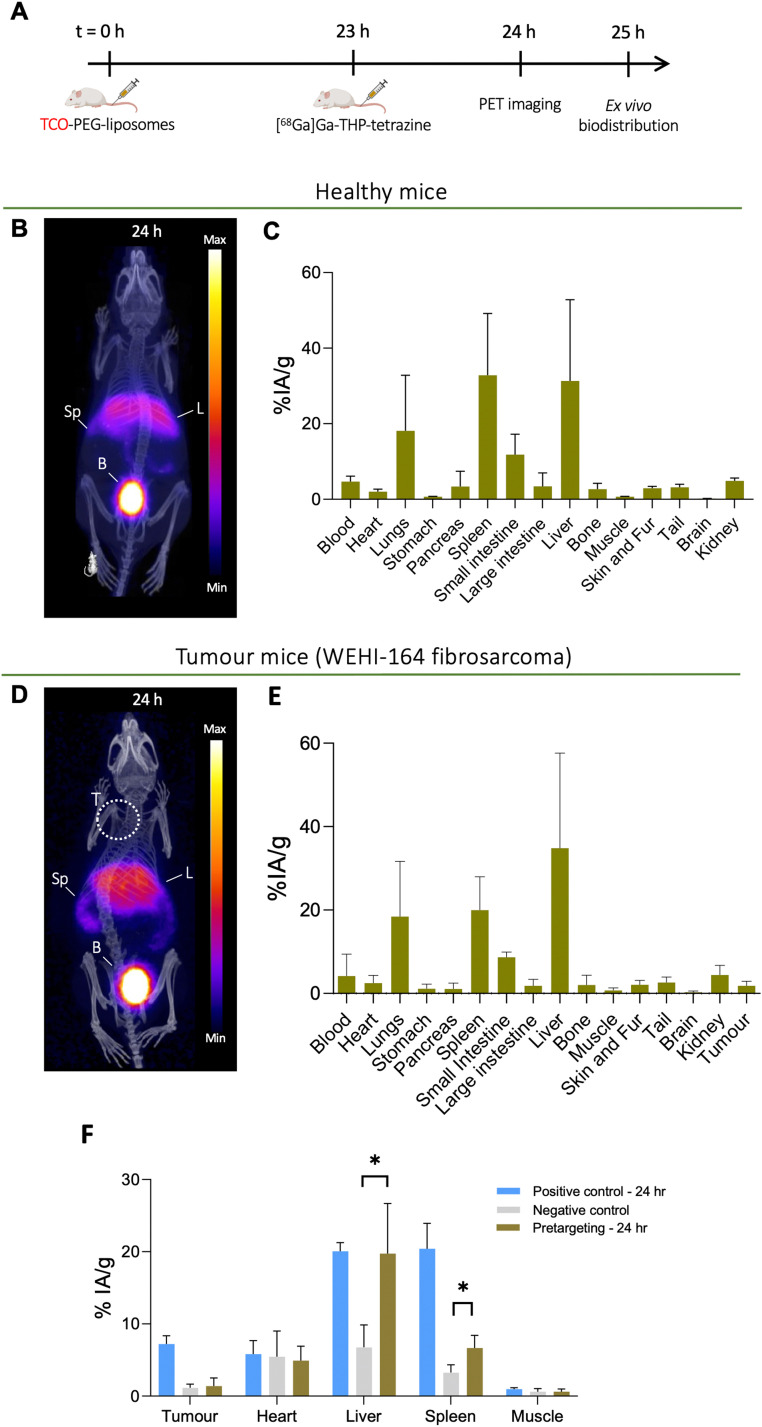
*In vivo* bioorthogonal pretargeting of TCO-PEG-liposomes in healthy and fibrosarcoma WEHI-164 tumour-bearing mice: (A) *in vivo* pretargeted imaging protocol schematics involving administration of TCO-PEG-liposomes followed by [^68^Ga]Ga-THP-Tz; (B) PET-CT image of a representative healthy mouse administered with TCO-PEG-liposomes at *t* = 0 h followed by administration of [^68^Ga]Ga-THP-Tz at *t* = 23 h and PET scan at *t* = 24 h (C) *ex vivo* biodistribution (*n* = 4) at *t* = 25 h of healthy mice administered with TCO-PEG-liposomes at *t* = 0 h followed by administration of [^68^Ga]Ga-THP-Tz at *t* = 23 h; (D) PET image of fibrosarcoma WEHI-164 tumour bearing mouse administered with TCO-PEG-liposomes at *t* = 0 h followed by administration of [^68^Ga]Ga-THP-Tz at *t* = 23 h and PET scan at *t* = 24 h; (E) *ex vivo* biodistribution at *t* = 25 h (*n* = 5) of fibrosarcoma WEHI-164 tumour bearing mouse administered with TCO-PEG-liposomes at *t* = 0 h followed by administration of [^68^Ga]Ga-THP-Tz at *t* = 23 h; (F) comparison of the image-based quantification measurements of the different experimental groups at *t* = 24 h. Statistical tests are between the pretargeting group and the negative group (*P* < 0.05).

Based on the positive control groups described above, the pretargeting test group was designed to track TCO-PEG-liposomes *in vivo* in the tumour, liver and spleen with a short-lived isotope which is desirable for tracking long-circulating nanomedicines and antibodies while minimising the radiation dose and non-specific binding. The general protocol for these studies is shown in Fig. 9(A). To optimise the timing of the injection of [^68^Ga]Ga-THP-Tz, the pretargeted imaging was initially performed in healthy animals at two time points: 4 h (see ESI,† Fig. S7) and 24 h post liposomal injection (Fig. 9(B) and (C)). At 4 h, the PET images showed radioactivity uptake in the urinary bladder, gall bladder, intestine, liver and spleen. The high urinary bladder, gall bladder and intestine accumulation observed is representative of the observed biodistribution of [^68^Ga]Ga-THP-Tz (negative control, see Fig. 4(B)) showing the clearance of the unreacted [^68^Ga]Ga-THP-Tz (PET images and biodistribution data in *in vivo* section in ESI†). The positive control showed high activity from the blood pool *i.e.* at 4 h p.i. (12 ± 2% IA g^−1^), as expected for long-circulating liposomes. However, this was not observed in the pretargeting group results with a blood pool (heart) uptake value at the earlier time point of 3.9 ± 0.7% IA g^−1^ (comparison shown in Fig. S7, ESI†). This result strongly suggests that blood-circulating liposomes were not labelled. The uptake observed in the liver and spleen is higher compared to the negative control showing validation of bioorthogonal pretargeting and the ability to image the TCO-PEG-liposomes in these two organs. The image quantification results further supported this observation, showing higher accumulation in the liver and spleen compared to the negative control images, but lower than the positive control (Fig. S7, ESI†). At *t* = 24 h (Fig. 9(B) and (C)), the pretargeting group PET images showed major accumulation in the urinary bladder, liver and spleen with minor uptake also in the lungs and intestines. The uptake observed in the lungs, bladder and intestines is expected due to the unreacted [^68^Ga]Ga-THP-Tz. However, the high uptake in the liver and spleen indicates biorthogonal pretargeted radiolabelling of the TCO-PEG-liposomes at 24 h. These observations were also confirmed by the biodistribution and image quantification data. However, the liver and spleen uptake values observed for the positive control (spleen: 48.8 ± 2.6% IA g^−1^ and liver: 44.4 ± 4.7% IA g^−1^) were higher than uptake values in the pretargeting test group (spleen: 32.8 ± 16.3% IA g^−1^ and liver: 31.3 ± 21.5% IA g^−1^). Also, the observed uptake values for the liver, spleen and lungs were highly variable which could be explained by the slightly lipophilic nature of the [^68^Ga]Ga-THP-Tz.

**Fig. 10:**
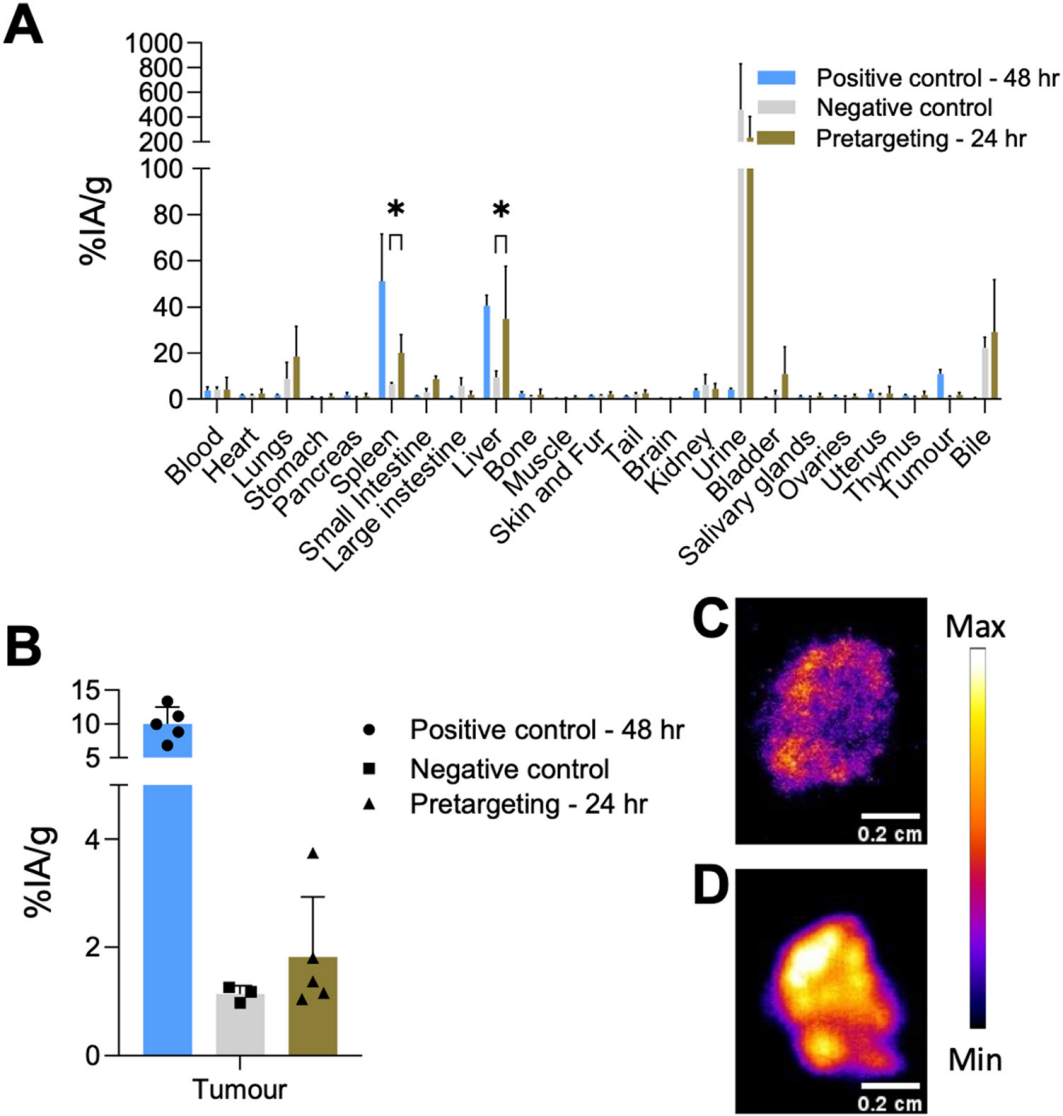
*Ex vivo* comparative analysis among different groups of the pretargeting tumour study. Note different timings for the pretargeting and positive control groups. (A) Comparison of the biodistribution of the different experimental groups. Statistical tests are between the pretargeting group and the negative group only, demonstrating that the observed uptake in the liver and spleen is statistically higher in the pretargeting group (*p* < 0.05); (B) comparison of the tumour uptake values observed for negative control and the pretargeting test group. Differences observed in tumour uptake values for the groups were non-significant; (C) autoradiography of tumour obtained from pretargeting test group showing low levels of radioactivity and hetereogenous distribution within the tumour tissue; (D) autoradiography of tumour obtained from the positive control group showing high levels of radioactivity and hetereogenous accumulation within the tumour tissue.

For the tumoured mice studies, the pretargeting experiment was only performed at one time point to minimize the potential saturation of TCO sites on the liposomes (Fig. 9(D)–(F)). THP-Tz was administered 24 h post administration of the TCO-PEG-liposomes. This time point was chosen due to two reasons: (i) the positive control showed significant tumour uptake (7.2 ± 1.1% IA g^−1^ from image analysis Fig. 7(B)) at *t* = 24 h enabling visualisation of pretargeting at the tumour; and (ii) to minimise the possible internalisation of liposomes accumulated at the tumour site. The PET images (Fig. 9(D)) and *ex vivo* quantification (Fig. 9(E)) showed high uptake in the liver and spleen as expected due to successful pretargeting in these organs, and in agreement with the previously discussed healthy animal group. Quantification of the image-based data at 24 h p.i. in comparison with the control groups (Fig. 9(F)) also revealed high uptake in the liver and spleen, compared to the negative control group (*p* < 0.05), and comparable to the positive control group. However, PET imaging and the image-based quantification studies revealed that there was no accumulation in tumours. The *ex vivo* biodistribution measurements at *t* = 25 h (Fig. 10(A)), showed a tumour uptake of 1.8 ± 1.1% IA g^−1^ which is significantly lower compared to that found with the positive control group (10.8 ± 2.0% IA g^−1^). However, the pretargeting group study was terminated at 24 h for *ex vivo* studies and the positive control study with [^67^Ga]Ga-THP-PEG-liposomes at 48 h. Thus, direct comparisons between these two groups cannot be made. Taken together, these results indicate that the bioorthogonal reaction has not occurred at the tumours. Compared to the uptake in tumours in the negative control, the tumour uptake in the pretargeting group is not-significantly different (Fig. 10(A) and (B)). This could be attributed to multiple factors, including the internalisation of the TCO-PEG-liposomes making them unavailable for binding, slow kinetics of the bioorthogonal reaction due to low TCO/tetrazine relative concentration, low tumour perfusion of [^68^Ga]Ga-THP-Tz or the isomerisation of the transcycloctene *in vivo* leading to the loss of reactivity towards tetrazine.

The tumours were collected during the *ex vivo* biodistribution studies for both the pretargeting and positive control groups, and fixed in OCT embedding fluid followed by autoradiography of 20 μm tumour sections (Fig. 10(C) and (D)). Both sections showed heterogeneous radioactivity distribution within the tumour tissue, with higher overall levels of activity in the positive control group (Fig. 10(D)) compared to the pretargeting group (Fig. 10(C)), as expected based on the *ex vivo* quantification results.

The development of a successful pretargeted system relies strongly on the elimination properties of the radiotracer of choice. Ideally, elimination through the renal clearance pathway is favoured over the hepatobiliary excretion, to minimise background radioactivity due to the long residence time of radiotracer in the liver and gut. In our case, the high uptake levels observed for [^68^Ga]Ga-THP-Tz in the intestines, gall bladder and liver complicate the delineation of nearby organs. The addition of a PEG linker between the tetrazine and THP fragments could be a strategy to modify and improve the pharmacokinetic profile of the radiotracer.^47^ The various tetrazines utilised for pretargeting of antibodies as well as nanoconstructs show the impact of different structural modifications on their bioorthogonal reactivity, pharmacokinetics in the body and elimination pathway.^40,42,48–51^ [^68^Ga]Ga-THP-Tz does show clearance through both renal and hepatobiliary excretion but allows for clear delineation of organs that are important in liposome clearance such as spleen, liver and tumours. However, it could be further improved to minimise hepatobiliary clearance. This could be achieved by decreasing its lipophilicity by using a PEG linker, a strategy that has been explored previously (*e.g.* log *D*_7.4_ [^68^Ga]Ga-DOTA-PEG_4_-Tz: −1.45, [^68^Ga]Ga-DOTA-PEG_11_-Tz: −2.19 ± 0.03)^23,38^ A lower lipophilicity might also alleviate the issue found with THP-tetrazine attaching to the vials and forming a lipophilic impurity on radiolabelling, thereby allowing us to avoid an extra purification step and maximising the amounts of tetrazine available for further *in vitro*/*vivo* applications.

Another important aspect to note is the effect of the chemical structure modification of tetrazine-based tracers on their reactivity towards TCO. A recent study by Steen *et al.* showed that the presence of different EWG and EDG has a major impact on the reactivity of tetrazine towards TCO.^40^ We chose the methyl tetrazine variant due to its stability in an aqueous buffer. However, this electron-donating methyl group on diene tetrazine leads to a decrease in the reactivity of tetrazine towards TCO which could be another possible explanation for the low pretargeting observed at the tumour. In future experiments, it would be interesting to examine a library of different tetrazine-THP compounds and their impact on their reactivity in the model system presented here.

## Conclusion

We have developed a new ^68/67^Ga-labelled tetrazine (THP-tetrazine) radiotracer with simple radiochemistry for bioorthogonal chemistry. In addition, we have also developed a TCO–phospholipid (TCO–PL) conjugate for reaction with ^68/67^Ga-THP-Tz based radiotracers which was successfully incorporated into the bilayer of preformed PEGylated liposomes to provide TCO-PEG-liposomes. These two components: [^68/67^Ga]Ga-THP-tetrazine and TCO-PEG-liposomes, provided a method for conventional and pretargeting imaging of PEGylated liposomes *in vivo* and *in vitro* with minimal impact on liposomal properties. The pretargeted nuclear imaging was performed in both healthy and fibrosarcoma tumour animals (syngeneic). The *in vitro* pretargeting experiments using this system were successful, even in the presence of competing serum proteins. However, the pretargeting results *in vivo* showed mixed results. The pretargeting of PEGylated liposomes was observed in healthy animals by showing efficient *in vivo* radiolabelling of the liposomes in the liver and spleen, but not of those that were circulating in the bloodstream. The pretargeting in tumour animals was also observed in the liver and spleen, but limited pretargeting was observed in the tumours. These results highlight both the potential advantages and drawbacks of bioorthogonal chemistry based on TCO and Tz agents, as the tetrazine conjugate was able to bind *in vivo* to some, but not all liposomes present in the animal; particularly those in blood as they should in principle be more accessible. Further work should include the pharmacological optimisation of this TCO/tetrazine pretargeting system to provide higher levels of *in vivo* radiolabelling and enhance binding of the radiotracer to blood-circulating biomolecules and important target organs such as the tumour.

## Experimental procedures

### Materials and methods

All inorganic and organic chemicals were of the highest purity grade available and used as received from Sigma Aldrich, Merck, CheMatech or Stratech. Deionised water treated with Chelex-50 resin was used for all metal-free chelation reactions. Plain HSPC/choline/mPEG2000-DSPE-liposomes were obtained from FormuMax Scientific Inc., USA. Gallium-68 was eluted as [^68^Ga]GaCl_3_ from an Eckert & Ziegler ^68^Ge/^68^Ga generator in ultra-pure HCl (5 mL, 0.1 M) manufactured to good manufacturing practice (GMP) requirements (ABX, Germany). Gallium-67 was obtained from Guy's radiopharmacy as [^67^Ga]Ga-citrate (5.5 mL, 600 MBq) manufactured for clinical use in patients. Nuclear magnetic resonance (NMR) data were acquired on a Bruker 400 MHz and analysed using MestReNova software. High-resolution mass spectrometry was performed on Autoflex, Bruker Daltonics at the Mass spectrometry facility at Waterloo campus, King's College London or the National Mass Spectrometry facility at Swansea on a Bruker ultrafleXtreme MALDI-TOF/TOF instrument. RadioHPLC experiments were performed on an Agilent 1260 Infinity instrument with ultraviolet (UV) detection at wavelength 254 nm and radioactivity detection using a Lablogic Flow-Count system with Bioscan Inc. B-FC-3200 photomultiplier tube detector. Liquid chromatography/mass spectrometry (LC/MS) data were acquired on an Agilent 1200 Series Liquid Chromatograph with UV detector at 254 nm, interfaced with an Advion Expression CMS mass spectrometer with electrospray ionisation (ESI) source. Preparative HPLC was performed on Agilent Technologies Prostar instrument interfaced with 410 Autosampler and 440-LC fraction collector. Radio ITLC was developed on Agilent Technologies glass microfibre chromatography paper impregnated with silicic acid. Radio instant thin-layer chromatography (ITLC) samples were recorded using a Lablogic Flow-count TLC scanner and a BioScan B-FC-3200 PMT detector and analysed using Laura software. Size exclusion chromatography (SEC) was performed on a Superose 10/30 column (GE Healthcare Life Sciences) run at 0.5 mL min^−1^ in PBS with UV detection performed at 214 and 280 nm on a GE Purifier ÄKTA HPLC. Manual SEC purification was PD MiniTrap^TM^ G-25 Medium size exclusion column containing 2.1 mL Sephadex resin (GE healthcare). Radioactivity for all samples was measured in a gamma counter (LKB Wallac 1282 Compugamma S) using EdenTerm software or dose calibrator (Capintec. Inc.). The centrifuge used was a Hettich MIKRO 20. Lyophilisation of purified samples was performed using an Edwards Freeze Dryer Modulyo. Hydrodynamic size and zeta potential were measured on Zetasizer Nano ZS from Malvern Panalytical. The electron microscopy facility at Imperial College London was used to screen the liposome samples under CryoEM. Preclinical imaging was performed in either preclinical NanoPET/CT imaging system (1:5 coincidence mode; 5-ns coincidence time window) or a NanoSPECT/CT imaging system (Aperture 3:1.2 mm multi-pinhole, frame time: 83 s, scan time ≈ 1 h) (Mediso Medical Imaging Systems, Budapest, Hungary). Autoradiography was performed on a cyclone phosphor imaging Typhoon system (Cytiva).

### Synthesis and characterisation of THP-tetrazine

6-Methyl tetrazine amine (4.5 mg, 0.021 mmol) was dissolved in DMSO (1 mL) followed by addition of THP-Bz-NCS (tris(hydroxypyridinone) isothiocyanate) (10 mg, 0.01 mmol). 20 μL of DIPEA (diisopropyl ethylamine) was added to the reaction, stirred at RT overnight and monitored using LCMS. The reaction mixture was purified using semi-prep HPLC method 1 (in ESI†) Pure THP-tetrazine collected was freeze-dried to give a pink fluffy solid (6.2 mg, 52% yield). (^1^H NMR in CD_3_CN/D_2_O (400 MHz) *δ* (ppm) 8.43–8.41 (m, 2H), 7.25–7.58 (6H), 6.96 (m, 6H), 4.89 (s, 3H), 4.54 (s, 2H), 3.79–3.82 (m, 17H), 3.01 (s, 2H), 2.48 (s, 3H), 2.42–2.44 (m, 2H), 2.11–2.15 (m, 4H), 1.95 (m, 9H), 1.85 (s, 2H), 1.25 (d, 4H)). ESI-mass spectrometry: (*m*/*z* [M + 2H]^2+^ = 581.52) and (*m*/*z* [M + H]^+^ = 1162.5).

### Radiochemistry

^68^Ga was eluted from ^68^Ge/^68^Ga generator with 0.1 N HCl, neutralised and purified to remove colloids as mentioned in ESI.† [^67^Ga]Ga–citrate was obtained from Guy's radiopharmacy and converted to [^67^Ga]GaCl_3_ for radiolabelling. The conversion was performed by following a modified protocol reported elsewhere.^52^

A THP-tetrazine stock solution (1 mg mL^−1^) was prepared in 10% DMSO/water and used for radiolabelling. THP-tetrazine (5 μL) was added to neutralised ^68/67^Ga (50–200 μL, ≈25 MBq) and incubated at R.T. for 15 minutes in a glass vial. The reaction mixture was spotted on an ITLC developed in either citrate buffer (0.175 M citric acid and 0.325 M trisodium citrate in water pH 5.5, Unbound ^68^Ga *R*_f_ = 0.7–1; [^68^Ga]Ga-THP-Tz *R*_f_ = 0; ^68^Ga colloid *R*_f_ =0), ammonium acetate: methanol (1 M, methanol concentration 80% v/v, unbound ^68^Ga *R*_f_ = 0; [^68^Ga]Ga-THP-Tz *R*_f_ = 0.7–1; [^68^Ga]Ga-THP-Tz colloid/impurity *R*_f_ = 0). Reaction purification was further performed using SEP-PAK plus light C18 cartridge and [^68^Ga]Ga-THP-Tz obtained post-purification was dried and resuspended in 5% DMSO/water.

The serum stability [^68^Ga]Ga-THP-Tz was performed in 50% (v/v) aqueous pooled serum from human male AB plasma. Samples (250 μL, 2–5 MBq) were added to serum (250 μL, filtered through a 0.22 μm filter) at a final volume (500 μL), and subsequently incubated at 37 °C in an Eppendorf vial heating block. Aliquots were taken after 0, 90, and 180 min and were analysed by HPLC method 4 (For HPLC method refer to ESI†).

### Synthesis and characterisation of TCO-PEG-liposomes

The insertion was performed by co-incubation of the PEGylated liposomes (400 μL, 60 mM lipid concentration) and the TCO–phospholipid dispersion (0.2 mg, 100 μL 10% ethanol/water) at 50 °C for 40 minutes under vigorous shaking. The formed TCO-PEG-liposomes are purified using PD Minitrap™ G-25 size exclusion column (SEC method in ESI†). Dynamic light scattering quantified the change in hydrodynamic size, zeta potential and PDIs of the PEGylated liposomes pre and post-insertion reaction. The concentration and the hydrodynamic of synthesised TCO-PEG-liposomes were measured by Nanoparticle tracking analyser. Cryoelectron microscopy was performed to determine the shape and size of the TCO-PEG-liposomes (additional information in ESI†).

### Radiochemistry

[^67/68^Ga]Ga-THP-Tz purified post radiolabelling was utilised for radiolabelling of TCO-PEG-liposomes. [^67/68^Ga]Ga-THP-tetrazine (100 μL, 5-20 MBq, 5% DMSO/water) was added to TCO-PEG-liposomes (100 μL, 50 mM lipid) and incubated at R.T. for 60 minutes. The radiolabelled TCO-PEG-liposomes were purified using PD Minitrap™ G-25 size exclusion column (Size exclusion purification method ESI†).

For serum stability, [^67^Ga]Ga-THP-PEG-liposomes were incubated at 37 °C in human serum in a 1 : 1 volume ratio of the radiolabelled liposomes to serum. Aliquots of the test sample were applied to SEC HPLC at 0 h, 24 h and 48 h and the radioactivity associated with the liposomes was determined.

### *In vitro* pretargeting methods

TCO-PEG-liposomes (200 μL, 4 mM) was added to either human serum (500 μL) or PBS (500 μL) and kept under incubation at 37 °C for 60 minutes. [^68^Ga]Ga-THP-Tz (100 μL, 1–4 MBq) was added to serum or PBS incubated TCO-PEG-liposomes and incubated at 37 °C. The incubated samples were purified using the size exclusion chromatography (refer to ESI† for SEC method). The liposome associated radioactivity and size exclusion column retained radioactivity was measured and bioorthogonal reaction was calculated using below:
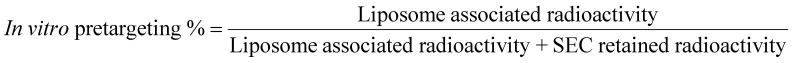


### *In vivo* pretargeting methods

All animal experiments were ethically reviewed by the Animal Welfare & Ethical Review Board at King's College London and carried out in accordance with the Animals (Scientific Procedures) Act 1986 (ASPA) UK Home Office regulations governing animal experimentation. The healthy and tumour animal studies were performed under PPL PBBA9A243 and P9C94E8A4 respectively. All *in vivo* experiments were conducted on healthy, female Balb/c mice (8–9 weeks old) obtained from Charles River UK Ltd. The cancer model experiments were also performed on BalB/c mice (8–9 weeks old) which were subcutaneously inoculated with mouse fibrosarcoma tumour cells.

For the subcutaneous tumour cell implantation, fibrosarcoma cell line (WEHI 164) syngenic to BALB/c mice was obtained from ATCC (batch number: CRL-1751TM). The tumour cells were subcutaneously implanted in anaesthetised mice over the shoulder in the following concentration: WEHI-164 (2.5 × 10^6^ cells per mL in 0.1–0.2 mL PBS). The mice were ready for imaging 8–10 days post tumour implantation. The mice chosen for imaging experiments had tumour sizes in the range of 70–100 mm^3^.

### Preclinical PET/CT and SPECT/CT scanners and biodistribution

Each mouse was anaesthetised by inhalation of isoflurane (2–3% in oxygen). The tracer/molecule of interest was injected *via* tail vein intravenous injection. The mice were scanned in either preclinical NanoPET/CT imaging system (1:5 coincidence mode; 5-ns coincidence time window) and NanoSPECT/CT imaging system (Aperture 3:1.2 mm multi-pinhole, frame time:83 s, scan time ≈1 h) (Mediso Medical Imaging Systems, Budapest, Hungary) depending on the tracer injected. (Refer to scanning section in ESI† for further details).

The mice were culled post-scan by cervical dislocation and required organs were collected, weighed, and measured for radioactivity using a Wallac gamma counter. Serial standard dilution of the injected radiotracer was measured alongside the collected organs to calculate the percentage injected dose (% IA g^−1^).

### *In vivo* imaging and biodistribution of THP-tetrazine

The THP-tetrazine was validated *in vivo* in healthy and tumour animals. For both healthy and tumour animals, the mice (*n* = 4) were anaesthetised and injected i.v. with [^68^Ga]Ga-THP-Tz (2–5 MBq, 100 μL). The mice were kept anaesthetised for 1-hour post-injection and then imaged *via in vivo* preclinical PET/CT imaging system. The mice were culled post scanning and biodistribution was performed.

### *In vivo* imaging and biodistribution of directly labelled [^67^Ga]Ga-THP-PEG-liposomes

Either tumour or healthy mice (*n* = 4) were anaesthetised and injected i.v. with [^67^Ga]Ga-THP-PEG-liposomes (3 MBq, 50 mM lipid concentration, 100 μL) and imaged at *t* = 4 h, 24 h *via* a preclinical SPECT-CT imaging system for healthy animals. For tumour studies, the animals were imaged at additional time points of *t* = 48 h to observe the tumour accumulation of liposomes at longer time points. The mice were culled post scanning and biodistribution was performed.

### Pretargeting of TCO-PEG-liposomes in healthy and tumour animals

For the pretargeting *in vivo* experiment in heathy animals, the mice (*n* = 4) were anaesthetised and injected i.v. with TCO-PEG-liposomes (50 mM lipid concentration, 100 μL) at *t* = 0 h. At *t* = 3 h, 23 h, mice were anaesthetised and injected i.v. with [^68^Ga]Ga-THP-tetrazine (2–5 MBq, 100 μL). For tumour studies, the mice (*n* = 4–5) were anaesthetised and injected i.v. with TCO-PEG-liposomes (50 mM lipid concentration, 100 μL) at *t* = 0 h. At 23 h, mice were anaesthetised and injected i.v. with [^68^Ga]Ga-THP-tetrazine (2–5 MBq, 100 μL). For both healthy and tumour studies, the mice were kept anaesthetised for 1-hour post-injection of radiotracer and then imaged *via in vivo* preclinical PET/CT imaging system. The mice were culled post-scan by cervical dislocation and required organs were collected, weighed, and measured for radioactivity along with the standards and *ex vivo* biodistributions were determined.

### Autoradiography

The tumours obtained from the positive control and test group were either snap frozen or formalin-fixed. The frozen/fixed tumours were submerged in OCT under liquid nitrogen and then cryosectioning into sections of thickness 20–30 μm. Autoradiography was performed to visualise the extent of delivery of prelabelled radioactive liposomes (positive control) and pretargeting (pretargeting test group) at the tumour. The obtained phosphor images were analysed using the free license software ImageJ.

## Data analysis

All numerical data were analysed on either GraphPad Prism 8 or advanced versions. Data are presented as mean ± standard deviation (SD) unless stated otherwise. All statistical tests performed are either multiple paired and unpaired *t*-tests.

The analysis of the reconstructed *in vivo* images was performed using the VivoQuant 3.5 (Invicro Inc.). For image quantification, regions of interest (ROI) were drawn over the knees for bones, heart as a measure of the blood pool, kidneys, lungs, bladder, spleen, liver, muscles, and brain in PET and SPECT images and the data from these ROIs was collected in %IA g^−1^ where %IA was determined by the amount of radioactivity administrated.

Additionally, for SPECT quantification, to determine radioactivity measured, a conversion from dimensionless image count values to units of radioactivity concentration in megabecquerel (MBq) units is made by the InVivoScope analysis software incorporating a user defined calibration factor stored within the software. This calibration factor was determined for gallium-67 and the 1.2 mm aperture (same aperture used for animal imaging) using a syringe filled with a known concentration of activity previously measured in a dose calibrator. The time of the measurement was also noted. The syringe was imaged acquiring a minimum of 100 000 counts per frame and the scan was reconstructed.

## Conflicts of interest

There are no conflicts of interest to declare.

## Supplementary Material

CB-005-D4CB00039K-s001
