# Supplementary material for: [68Ga]Ga-THP-tetrazine for bioorthogonal click radiolabelling: pretargeted PET imaging of liposomal nanomedicines
Source: RSC Chem Biol. 2024 May 14;5(7):622–39. doi: 10.1039/d4cb00039k (PMC11221536; doi:10.1039/d4cb00039k)
Supplement: CB-005-D4CB00039K-s001 [file CB-005-D4CB00039K-s001.pdf]

Electronic Supplementary Material (ESI) for RSC Chemical Biology.
This journal is © The Royal Society of Chemistry 2024

Supporting information for:

[^{68}Ga]Ga-THP-Tetrazine for bioorthogonal click radiolabelling: Pretargeted PET imaging of liposomal nanomedicines

Aishwarya Mishra[1], Amaia Carrascal-Miniño[1], Jana Kim[1],

Rafael T.M. de Rosales[1]

[1]School of Biomedical Engineering & Imaging Sciences, King's College London,

St Thomas' Hospital, London, SE1 7EH, UK

Table of contents

Contents

Section 1 Synthesis and Characterisation of THP-tetrazine......2

Section 2 Synthesis and characterisation of TCO-phospholipid......4

Section 3 Characterisation of TCO-PEG-liposomes......7

  1. Nanoparticle Tracking Analysis......7

  2. Cryo-Electron Microscopy......7

Section 4 Purification methods......7

Section 5 ^{68}Ga purification......10

Section 6 Log P and Log $D_{7.4}$ measurement......11

Section 7 Serum stability method......11

Section 8 PET/SPECT imaging reconstruction method......12

Section 9 *In vivo* pretargeted imaging at t = 4 hr time point......12

Section 10 In vivo biodistribution data......13

Section 11 Zeta potential measurement of ^{67}Ga-TCO-PEG-liposomes......15

Section 1. Synthesis and Characterisation of THP-tetrazine

To remove unreacted THP-Bz-SCN and excess reaction components (*i.e.,* DIPEA, DMSO), semi-prep HPLC (method 2) on a reverse phase column was utilised to provide the pure THP-tetrazine. The reaction was monitored by LCMS showing that the THP-tetrazine is collected in the fraction where the mobile phase is approximately between 40-50% acetonitrile/water. Using the information obtained from LCMS, we estimated that for HPLC purification, acetonitrile concentration higher than 25 % will be required to elute the THP-tetrazine product. Therefore, a slow gradient as shown in HPLC method 2 was applied with a semi-automated system. The THP-tetrazine was collected at t_r = 29 minutes whereas unreacted tetrazine was collected at t_r = 17 minutes as shown in figure S2(A). The purity of collected THP-tetrazine was verified using LCMS of the collected fractions showing THP-tetrazine (m/z $[M + 2H]^{2+}$ = 581.52) and (m/z $[M+H]^{+}$ = 1162.5) (figure S2(C)).

The isolated pure product was obtained in average yield (57 ± 5 %) as a bright pink hygroscopic solid and stored for later use at -20°C. Further characterisation and confirmation of the purity of the synthesised THP-tetrazine was obtained from ^{1}NMR spectrum in a mixture of CD_3CN/D_2O. The

characteristic peaks of the THP-tetrazine observed were δ 8.43-8.41 (d, (J = 8.3 Hz), 2H Ar-H from tetrazine), 7.57 (d, (J = 8.2 Hz), 2H)7.25-7.36 (4H from N-H), 6.96 (m, 6H from Ar-H from tetrazine), 4.89(s, 3H from Ar-H from THP), 4.54 (s, 2H), 3.79-3.82 (m, 9H from CH_3-N, 8H from CH_2-N), 3.01 (s, 2H) 2.48 (s, 3H), 2-42-2.44 (m, 2H), 2.11-2.15 (m, 4H), 1.95 (m, 9H), 1.85 (s, 2H), 1.25 (m, 4H).(Figure S1) ^{1}H NMR (400 MHz, CD_3CN).

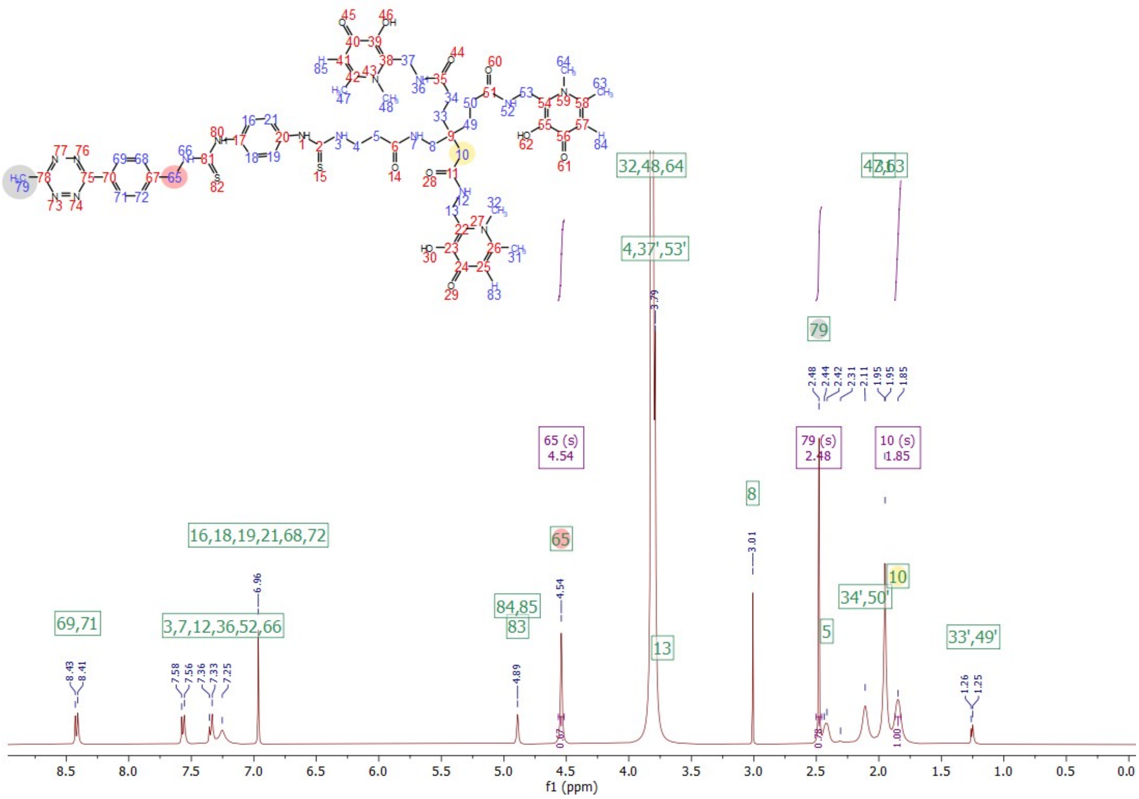

Figur
e S1. NMR spectra of the THP-tetrazine: The synthesised THP-tetrazine was characterised by the presence of the above peaks. The spectrum was recorded in CD_3CN/D_2O due to low solubility in $CDCl_3$. ^{1}H NMR (400 MHz) δ 8.43-8.41 (d, (J = 8.3 Hz), 2H Ar-H from tetrazine), 7.57 (d, (J = 8.2 Hz), 2H)7.25-7.36 (4H from N-H), 6.96 (m, 6H from Ar-H from tetrazine), 4.89(s, 3H from Ar-H from THP), 4.54 (s, 2H), 3.79-3.82 (m, 9H from CH_3-N, 8H from CH_2-N), 3.01 (s, 2H) 2.48 (s, 3H), 2-42-2.44 (m, 2H), 2.11-2.15 (m, 4H), 1.95 (m, 9H), 1.85 (s, 2H), 1.25 (m, 4H). Non-deuterated solvent H_2O and CD_3CN impurity peaks are also observed in the spectrum.

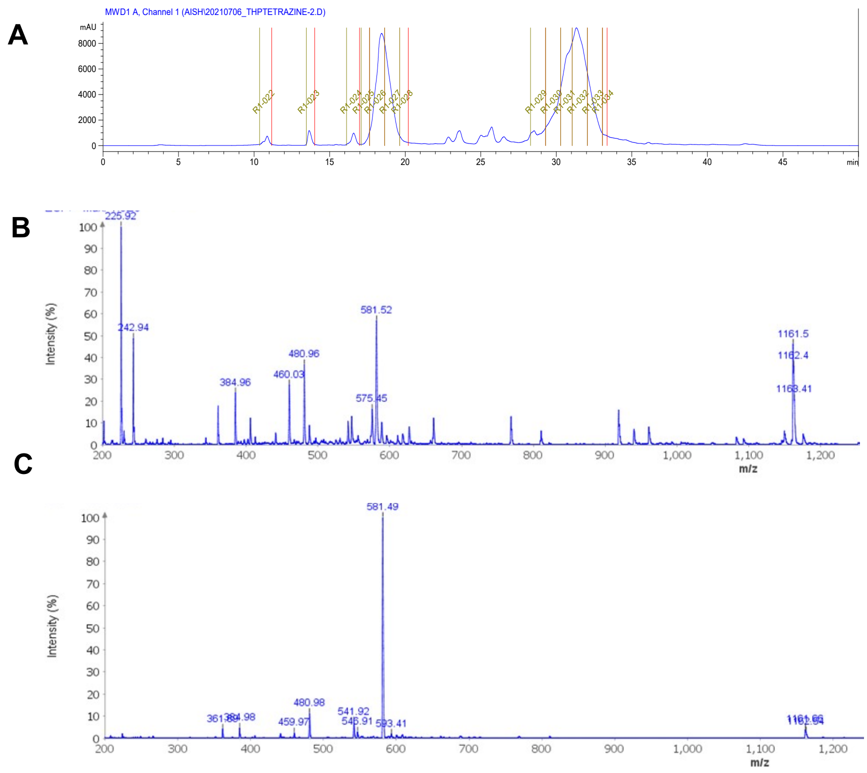

Figure S2. Purification of THP-tetrazine: (A) HPLC chromatogram of the semi-prep HPLC method used for isolation of THP-tetrazine from the reaction mixture. Fraction collected between t_r =29 min-33 min contained the THP-tetrazine; (B) Mass spectra of the THP-tetrazine crude reaction mixture showing the THP-tetrazine as the major product alongside unreacted Methyl-tetrazine amine and hydrolysed THP; and (C) Mass spectra of the THP-tetrazine fraction collected after semi-prep HPLC showing the pure isolated THP-tetrazine.

Section 2. Synthesis and characterisation of TCO-phospholipid

TCO-PEG$_4$-NHS ester (8.2 mg, 0.0159 mmol) was dissolved in DMSO (1 mL) followed by addition of DSPE-PEG (2000)-amine (15 mg, 0.0053 mmol). DIPEA (20 µL) was added to the reaction mixture and incubated at R.T. overnight. TCO-Phospholipid was purified using dialysis, lyophilised, weighed (reaction yield: 96%) and stored at -20°C for further use. ^{1}H NMR (400 MHz, CDCl$_3$) δ 6.66 (s, 1H), 5.57-5.53 (m, 2H), 5.17-5.14 (m, 1H), 4.67 (s, 1H), 4.23-4.06 (m, 6H), 4.18 (s, 2H), 3.76-3.47 (m, 196H), 3.40-3.37 (s, 2H), 3.28 (s, 2H), 2.69-2.66 (t, 2H), 2.55-2.52 (t, 2H), 2.42 (s, 1H), 2.27-2.21 (m, 4H), 2.10-2.01 (m, 4H), 1.89-1.84 (m, 6H), 1.74 (m, 2H), 1.70 (m, 2H), 1.19 (s, 56H), 0.81 (t, 6H))(Figure S3) . TCO-PL was also characterised by high-resolution mass spectrometry (TCO-PL: m/z=2700-3700 (z=1); DSPE-PEG2000 amine m/z=2400-3200 (z=1)).

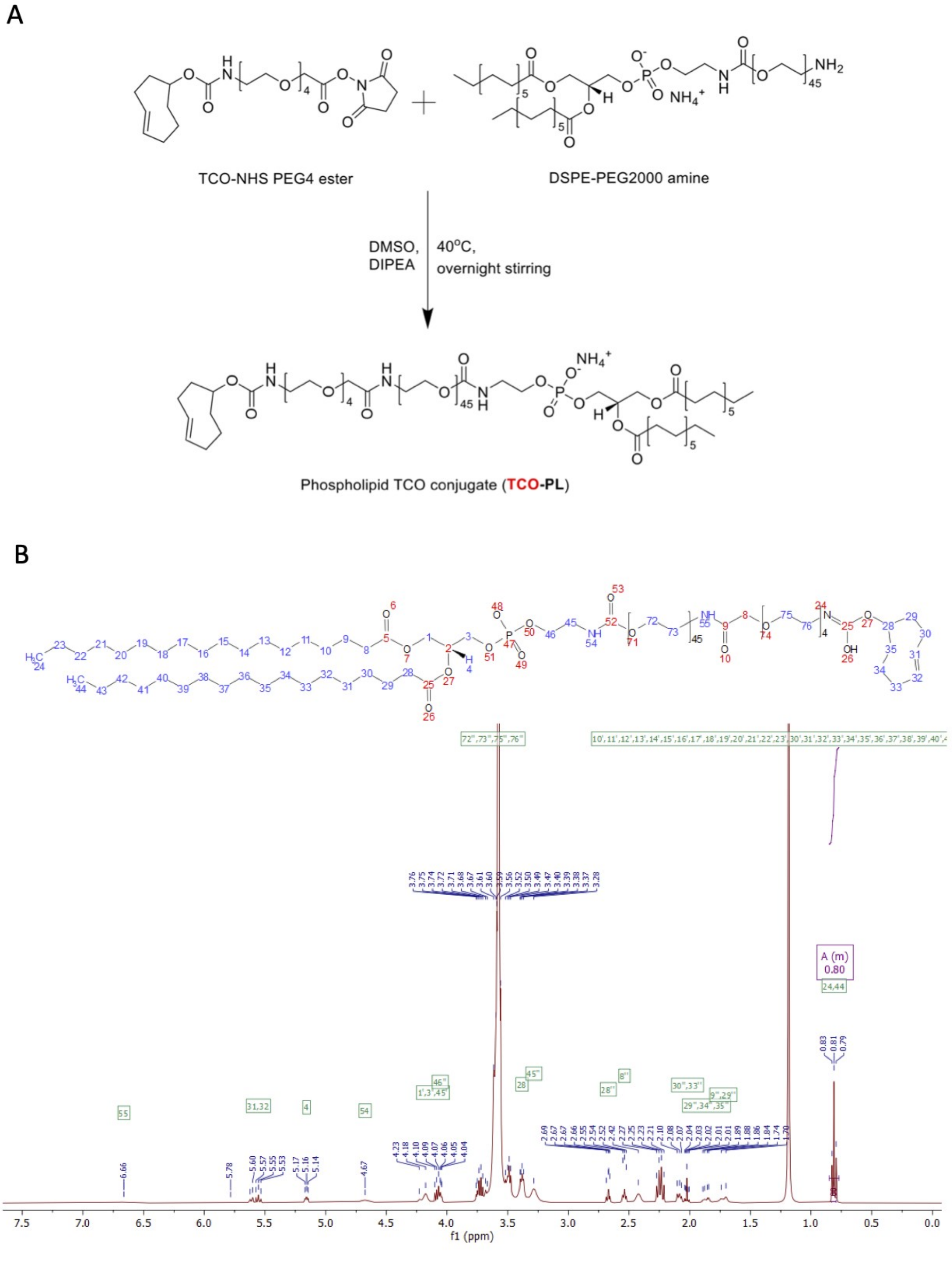

Figure S3. (A) Schematic representation of the synthesis of TCO-PL; (B) Characterisation of TCO-PL. NMR spectra of TCO-PL: The synthesised TCO-PL is characterised by the presence of characteristic peaks of TCO incorporated in the phospholipid: δ 2.5 -CH; 5.5 =CH in cyclooctene. The integration of the peak's accounts for 283 out of 293 protons. The integration is not quantitative due to the relative high intensity of protons from ethylene peak of the PEG2000 chain which decrease the resolution of other peaks.

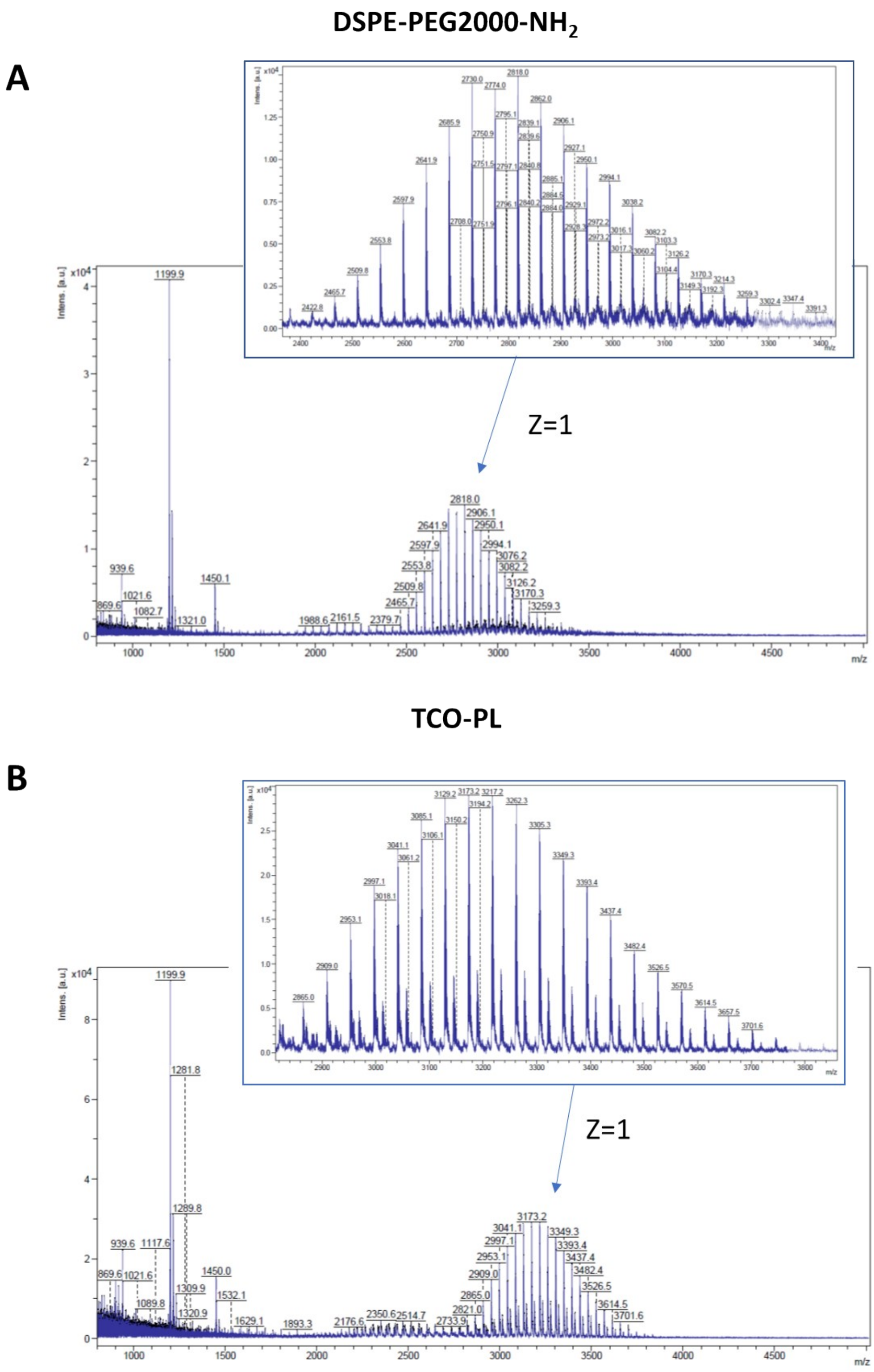

Figure S4. The mass spectrum of the TCO-PL (A) and DSPE-PEG2000 amine (B): The envelope nature observed in both spectra is due to the polydispersity of the PEG2000 chain which is characterised by a difference of 44 between each peak within the envelope. The TCO-PL is characterised by the higher m/z=2700-3700 compared to the lower m/z for DSPE-PEG2000 amine m/z=2400-3200.

Section 3. Characterisation of TCO-PEG-liposomes

1. Nanoparticle Tracking Analysis

The concentration and the hydrodynamic of synthesised TCO-PEG-liposomes were measured by NTA using NanoSight LM10 and NTA software v3.2 (Malvern Panalytical). The stock sample was diluted to achieve ~100 particles/viewing frame. Measurements were made in triplicates for 60 s with a 488 nm laser, for up to three serial dilutions of the sample.

2. Cryo-Electron Microscopy

QUANTIFOIL R 2/2 carbon grids (mesh: Cu 300, #234901; Agar Scientific) were plasma discharged for 50 s at 30 SCCM gas flow in Nanoclean 1070 (Fischione instruments). Aliquots (5 µL) of TCO-PL-liposomes or PEGylated liposomes were deposited on the carbon grids in Vitrobot Mark IV (FEI). The excess liquid was removed by blotting with filter paper (Agar Scientific); Parameters: blotting time = 2 s, wait time = 30 s, and blotting force = 2. The grids were instantly frozen in liquid ethane (−188 °C) and maintained in liquid N_2 (−196 °C) in a grid box and transferred into a cryo-transfer holder. CryoEM of these samples was recorded on TECNAI 12 G2 (FEI) system interfaced with a TemCam-F216 camera and operated using Temmenu v4 software (Tietz Video & Image Processing Systems GmbH, Germany). Parameters used to capture images are as follows: electron acceleration = 120 kV, magnification = 52,000×, acquisition time = 1 s, and spot size = 5.

Section 4 Purification methods

The following HPLC/FPLC methods were used:

Method 1. Analytical HPLC method for LC/MS for characterisation of THP-tetrazine
Solvent A= Water (0.1% Trifluoroacetic acid v/v), Solvent B= Acetonitrile (0.1% Trifluoroacetic acid v/v)

TIME (MIN)	FLOW RATE (ML MIN-1)	% A	% B
0	1	95	5
2	1	95	5
11	1	5	95
12	1	5	95
12.1	1	95	5
15	1	95	5

Column: Agilent Eclipse XDB-C18 column (4.6 x 150 mm, 5 µm)

Method 2. Semi-prep HPLC method for purification of THP-tetrazine
Solvent A= Acetonitrile (0.1% Trifluoroacetic acid v/v), Solvent B= Water (0.1% Trifluoroacetic acid v/v)

TIME (MIN)	FLOW RATE (ML MIN-1)	% A	% B
0	4	5	95
2	4	5	95
52	4	50	50
62	4	5	95

Column: Agilent Zorbax XDB C18 (21.2 x 150 mm, 5 µm)

Method 3. Analytical radioHPLC method for characterisation of [^{68}Ga]Ga-THP-tetrazine
Solvent A= Water (0.1% Trifluoroacetic acid v/v), Solvent B= Acetonitrile (0.1% Trifluoroacetic acid v/v)

Time (min)	Flow rate (mL min-1)	% A	% B
**0**	1	95	5
**2**	1	95	5
**11**	1	5	95
**12**	1	5	95
**12.1**	1	95	5
**15**	1	95	5
**Column: Agilent Eclipse XDB-C18 column (4.6 x 150 mm, 5 µm)**			

Method 4. Serum stability HPLC method
Solvent A = phosphate buffered saline

Time (min)	Flow rate (ml min^{-1})	Solvent A (%)
**0**	1	100
**40**	1	100
**Column: Phenomenex BioSep SEC-s2000 column (300 x 7.8 mm, 5 µm)**		

Method 5. Size exclusion purification method

A G-25 size exclusion column pre-equilibrated with 15 column volumes of 0.9% filtered saline was used. The test sample for purification is applied to the column in 500 µL volume, followed by 750 µL fractions which are collected in separate vials. Liposomes elute in the first 750 µL fraction as seen in figure below.

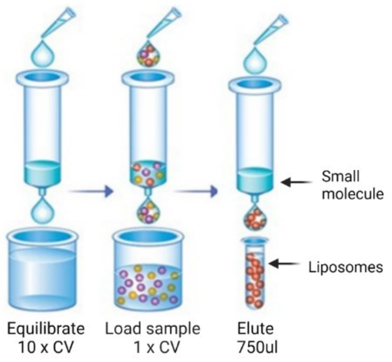

Figure S5. Procedure for purification of liposomes using size exclusion chromatography column. Created with BioRender.com.

Section 5. ^{68}Ga purification

All radioactive work was performed manually with all necessary radioprotection measures in place. ^{68}Ga was manually eluted from ^{68}Ge/^{68}Ga generator with 0.1 N HCl. The peak radioactivity containing 1 mL elution was used for radiolabelling after buffering with 3.4 M Sodium acetate/1 M Sodium carbonate to pH 6. Buffered ^{68}Ga was used for all radiolabelling and i.v. injections after removal of colloids using saline pre-rinsed centrifugal filter MW cutoff 50 kDa.

The colloid formation during neutralisation of generator eluate competes with the radiolabelling of the chelator and provides an impure final reaction mixture requiring a need for purification of the radiolabelling mixture post labelling. To minimise the presence of colloids, post buffering of generator eluate to pH 6, buffered Ga was purified using a centrifugal spin filter which removed any colloids formed. This was verified using radioTLC as seen in figure S6.

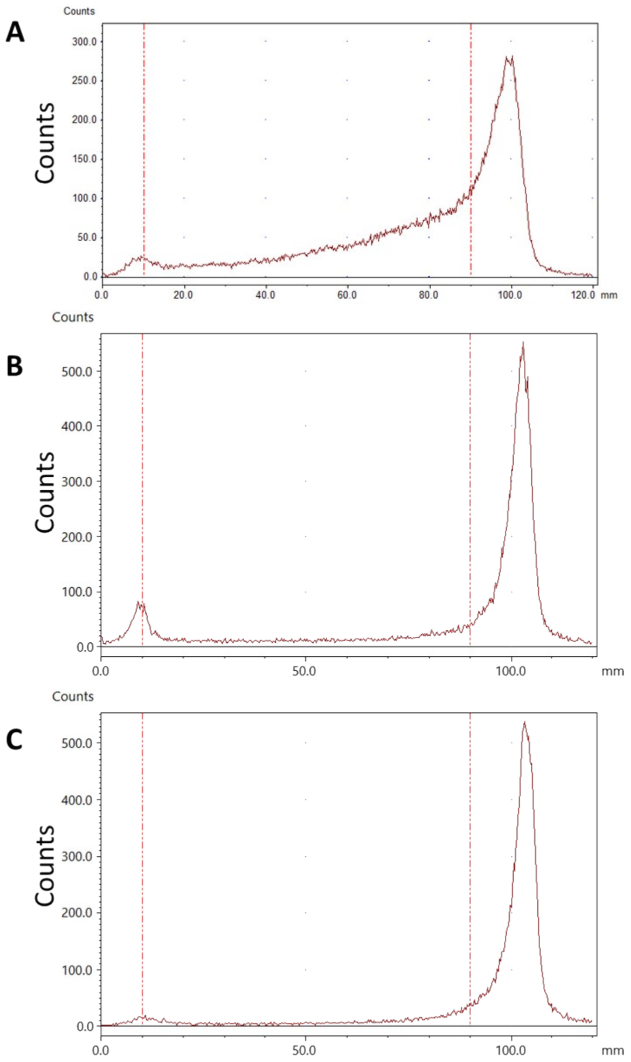

Figure S6. Purification of ^{68}Ga: The colloids generated after neutralisation of gallium generator eluate are removed and the different gallium samples pre and post purification are characterised using ITLC **(A)** Generator eluate; **(B)** Neutralised ^{68}Ga pre-purification and **(C)** Neutralised ^{68}Ga post-purification

Section 6 Log P and Log $D_{7.4}$ measurement

For log P measurement, [^{68}Ga]Ga-THP-Tz (50 µL, 1 MBq) were added to vials containing a mixture of water saturated octanol (500 µL) and octanol saturated water (500 µL). The tubes were shaken for 3 min and the mixture was centrifuged for 5 min to separate the octanol and water phases. Aliquots (50 µL) of each phase were taken and transferred to separate vials for counting of radioactivity. For log $D_{7.4}$ measurement, the same procedure was performed by replacing water with PBS. All experiments were performed in triplicates.

Section 7. [^{68}Ga]Ga-THP-tetrazine serum stability method

The stability of [^{68}Ga]Ga-THP-tetrazine was evaluated at 37˚C in commercially obtained human serum for up to 3 hours using HPLC SEC which separated THP-tetrazine and serum components. The serum protein bound ^{68}Ga eluted at t_r = 9 min whereas [^{68}Ga]Ga-THP-tetrazine eluted at t_r = 20 min when the serum incubated samples were analysed using serum stability HPLC method 4. The analysis of serum incubated samples showed minimal transchelation of ^{68}Ga to blood serum proteins and high stability of >95 % after 3 hours of incubation as shown in figure S7.

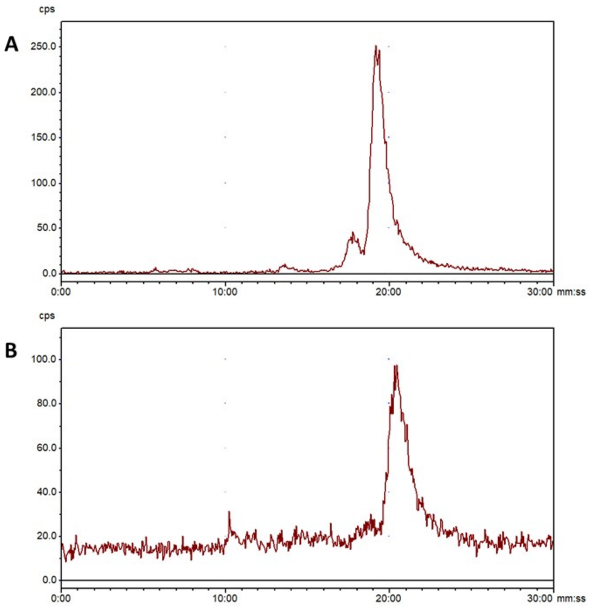

Figure S7. Serum stability assessment of [^{68}Ga]Ga-THP-tetrazine using HPLC SEC method 4: (A) RadioHPLC chromatogram of serum incubated [^{68}Ga]Ga-THP-tetrazine at t = 15 minutes; (B) RadioHPLC chromatogram of serum incubated [^{68}Ga]Ga-THP-tetrazine at t = 3 hours; RadioHPLC chromatogram species: serum bound ^{68}Ga (t_r= 10 min), [^{68}Ga]Ga-THP-tetrazine (t_r = 20 min).

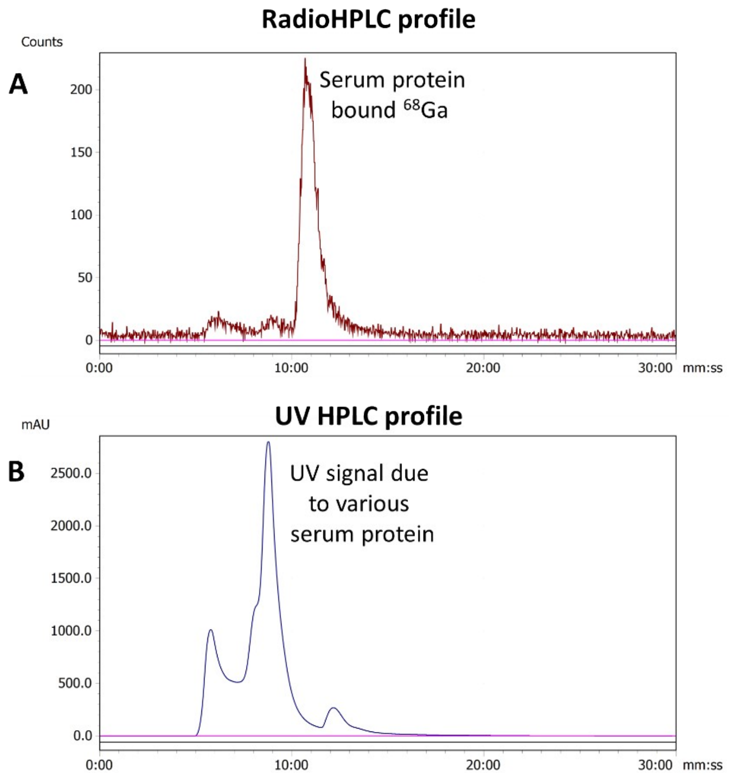

Figure S8. HPLC SEC control serum stability run: (A) RadioHPLC chromatogram showing the elution of serum bound ^{68}Ga; (B) UV chromatogram (280 nm) of proteins present in human serum.

Section 8. PET/SPECT imaging reconstruction method

PET/CT images were reconstructed using Tera-Tomo 3D reconstruction (400–600 keV energy window, 1–3 coincidence mode, 4 iterations and subsets) at a voxel size of (0.4 × 0.4 × 0.4) mm3 and corrected for attenuation, scatter, and decay. The data were binned into 17 frames (1 × 1, 10 × 3, 5 × 5 and 1 × 4 min) for dynamic analysis. SPECT images were reconstructed using HiSPECT standard method.

Section 9. *In vivo* pretargeted imaging at t = 4 hr time point

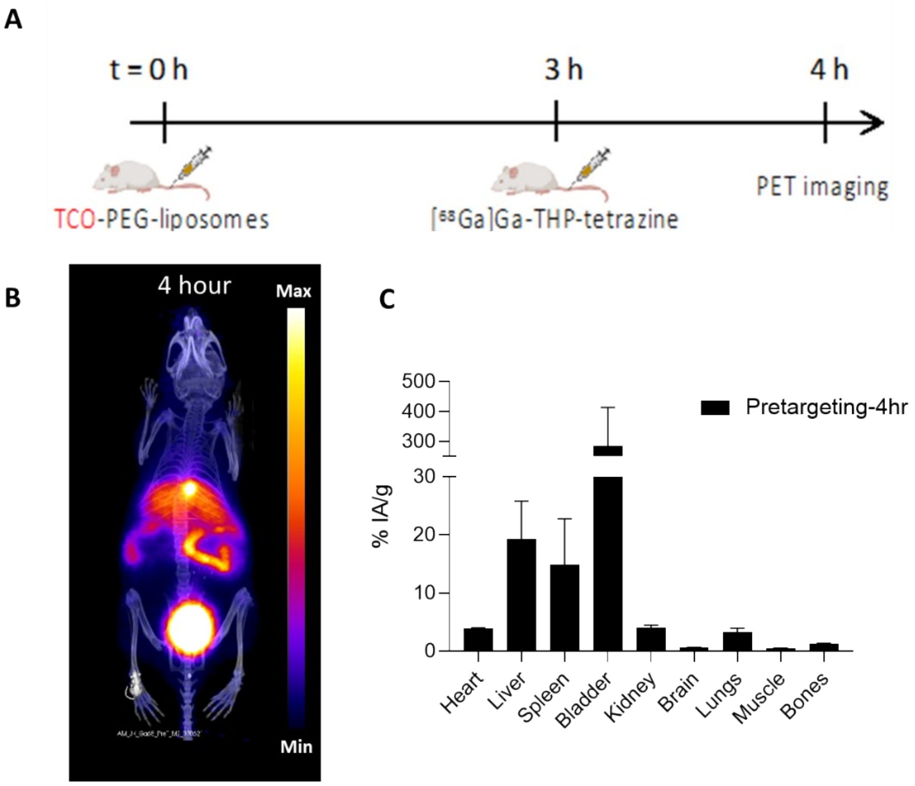

Figure S8. Pretargeting test *in vivo* group at t = 4 h(n=4): (A) Schematic of pretargeting test group; (B) PET image of animal administered with TCO-PL-liposomes at t= 0 h followed by administration of [^{68}Ga]Ga-THP-tetrazine at t = 3 h and PET scan at t = 4 h; (C) Image quantification performed on the PET images acquired at t = 4 h.

Section 10. *In vivo* biodistribution data

The tables below show the biodistribution data obtained from the *in vivo* experiments performed on this study.

Table S1. Biodistribution data from the TCO-PL-liposomes pretargeting experiment on healthy animals

	[68 Ga]Ga-THP-tetrazine (Negative control)			Pretargeting group-24 h			[67Ga]Ga-TCO-liposomes (Positive control)- 24 h		
	Mean	Standard dev	n	Mean	Standard dev	n	Mean	Standard dev	n
Blood	5.626807911	2.45921956	4	4.674034751	1.43091048	4	16.98061324	2.757014718	4
Heart	2.534111445	1.310687271	4	2.020845332	0.651391938	4	4.334614519	0.388103858	4
Lungs	28.03630434	7.66594347	4	18.12860383	14.70351432	4	5.780277022	0.8651312	4
Stomach	0.970225656	0.503065361	4	0.65832653	0.115264928	4	1.066092208	0.352535389	4
Pancreas	0.885373628	0.473348616	4	3.391809463	4.041000404	4	1.75344565	0.520053016	4
Spleen	11.87526429	6.027187305	4	32.82954566	16.3309688	4	48.8433591	2.629610051	4
Small intestine	21.30868063	6.874148889	4	11.8192986	5.424517069	4	2.220519374	0.184252849	4
Large intestine	2.033573358	1.585917596	4	3.411370967	3.577939566	4	1.298217835	0.084173876	4
Liver	22.37020567	11.44575327	4	31.30443392	21.54211264	4	44.45899745	4.784803357	4
Bone	1.327226797	0.465553275	4	2.676462914	1.569143175	4	3.489126499	2.145516357	4
Muscle	0.62218617	0.296595309	4	0.665588529	0.091346205	4	0.525962398	0.102649409	4
Skin and Fur	3.858789788	1.851406863	4	2.934304287	0.483974034	4	1.414591504	0.218278802	4
Tail	3.58191388	1.965527666	4	3.195798508	0.779530681	4	1.625103345	0.746248968	4
Brain	0.268754083	0.125391133	4	0.171779431	0.042736477	4	0.986483798	0.266561575	4
Kidney	6.752648032	5.079592266	4	4.886517577	0.730655011	4	5.831151214	0.749681987	4

Table S2. Biodistribution data from the TCO-PL-liposomes pretargeting experiment on tumour animals

	[67Ga]Ga-TCO-liposomes-48 h (Positive control)			[68Ga]Ga-THP-tetrazine (Negative control)			Pretargeting group- 24 h		
	Mean	Standard dev	n	Mean	Standard dev	n	Mean	Standard dev	n
Blood	3.745671	1.575558	4	4.210877	0.947796	3	4.168174	5.257386	5
Heart	1.553543	0.497039	4	1.553129	0.449273	3	2.450205	1.886517	5
Lungs	1.733273	0.330726	4	8.829553	7.099763	3	18.43101	13.22112	5
Stomach	0.738245	0.169509	4	0.652772	0.089637	3	1.122039	1.126008	5
Pancreas	1.55159	1.294178	4	0.753637	0.208766	3	1.052	1.418785	5
Spleen	51.02272	20.41804	4	6.502822	0.58481	3	19.99855	8.000653	5
Small Intestine	1.317335	0.151532	4	3.141064	1.383721	3	8.659665	1.267427	5
Large instestine	0.885356	0.204259	4	5.846163	3.318049	3	1.807485	1.571937	5
Liver	40.6665	4.389445	4	9.446526	2.739664	3	34.83466	22.79337	5
Bone	2.493422	0.739933	4	1.029953	0.472213	3	2.012306	2.351149	5
Muscle	0.278623	0.091204	4	0.440502	0.091305	3	0.711072	0.599797	5
Skin and Fur	1.516088	0.273866	4	1.497613	0.349109	3	2.08836	1.028997	5
Tail	1.175871	0.343965	4	2.091081	0.650515	3	2.623479	1.321404	5
Brain	0.15547	0.139344	4	0.145119	0.043302	3	0.282092	0.291367	5
Kidney	3.803221	0.696908	4	6.264872	4.499794	3	4.423072	2.285061	5
Urine	4.169576	0.508672	4	461.0004	366.8738	3	234.6064	169.7733	5
Bladder	0.547079	0.113357	4	1.879929	1.882884	3	10.82124	11.87311	5
Salivary glands	0.907305	0.60242	4	0.764007	0.346333	3	1.409566	1.042137	5
Ovaries	0.940056	0.653437	4	0.694487	0.465829	3	1.117211	1.065748	5
Uterus	2.701091	1.255419	4	1.609073	0.785208	3	2.448279	3.01738	5
Thymus	1.507303	0.547012	4	1.010553	0.090178	3	1.701703	1.744682	5
Tumour	10.83139	1.93873	4	1.135511	0.149272	3	1.8213	1.113882	5
Bile	0.346731	0.261945	3	22.38266	4.51	3	29.10507	22.56379	5

Section 11. Zeta potential measurement of ^{67}Ga-TCO-PEG-liposomes

Sample	Mean Zeta potential (in mV)	Std. dev
PEG liposomes	-0.722666667	0.338768849
TCO-PEG-liposomes	-1.683333333	0.217332311
^{67}Ga-TCO-PEG-liposomes	-1.507666667	0.860497724
